# National trends in bone-metastatic prostate cancer mortality in the United States, 1999–2024: a multiple-cause-of-death analysis with projections to 2040

**DOI:** 10.3389/fonc.2026.1882666

**Published:** 2026-07-20

**Authors:** Kanza Atif, Eshal Atif, Vishan Das, Alaa Eldeeb, Kaneez Fatima, Sadia Qazi, Aziz Abdullah

**Affiliations:** 1 College of Medicine, Alfaisal University, Riyadh, Saudi Arabia; 2 Liaquat University of Medical and Health Sciences, Jamshoro, Sindh, Pakistan; 3 Faculty of Medicine, Alexandria University, Alexandria, Egypt; 4 CMH Institute of Medical Sciences, Multan, Pakistan; 5 Department of Anatomy, College of Medicine, Alfaisal University, Riyadh, Saudi Arabia; 6 Department of Urology, Liaquat National Hospital and Medical College, Karachi, Pakistan

**Keywords:** bone metastasis, cancer surveillance, CDC WONDER, joinpoint regression, mortality trends, multiple cause of death, prostate cancer, racial disparities

## Abstract

**Background:**

Conventional underlying-cause surveillance shows a declining prostate cancer mortality trend in the United States. MCOD analysis, which also includes deaths where bone metastasis was co-documented as a contributing cause, may identify a different trajectory than single-cause coding. Objective: To characterize 25-year trends in prostate cancer mortality with co-documented bone metastasis, quantify divergence from underlying-cause surveillance, and project age-adjusted mortality rates (AAMRs) to 2040 across demographic and geographic subgroups.

**Methods:**

We queried CDC WONDER (1999–2024) for deaths with prostate cancer (ICD-10: C61) as the underlying cause and bone metastasis (C79.5) as a contributing cause. Joinpoint regression characterized temporal trends, and ARIMA and exponential smoothing models with RMSE/MAPE-based selection generated stratum-specific projections. Analyses were stratified by age, race/ethnicity, US Census region, and urbanization.

**Results:**

In total, 73,429 deaths met the dual-code definition. The overall AAMR rose 93%, from 1.10 to 2.12 per 100,000 [average annual percent change (AAPC): +2.61%; 95% CI: 1.66–3.56; p < 0.000001]; however, this net increase followed an initial decline from 1999 to 2006 [annual percent change (APC): −5.57%] and reflected an acceleration to +12.91% annually during 2013–2018. Conventional underlying-cause surveillance showed an AAPC of −1.55%. Non-Hispanic Black or African American men had a mean AAMR 64% higher than non-Hispanic White men (1.89 vs. 1.15 per 100,000); in model-based projections, this ratio widened from 1.54 (2025) to 1.78 (2040). In 2024, men aged 65–85+ had a 17-fold higher AAMR than those aged 45–64. The overall AAMR is projected to reach 3.65 (95% CI: 0.88–6.43) by 2040, with wide prediction intervals reflecting substantial uncertainty.

**Conclusions:**

MCOD surveillance identified a sustained 25-year increase in co-documented prostate cancer and bone-metastasis deaths, diverging from the declining underlying-cause-only trend. The post-2013 acceleration predates the COVID-19 pandemic and persisted in sensitivity analyses. Rates were higher among older men, non-Hispanic Black or African American men, and non-metropolitan residents. As death-certificate–based measures, these estimates reflect documented bone metastasis rather than the true incidence of bone-metastatic disease and may be affected by evolving documentation and coding practices. Findings support incorporating MCOD-based metrics into national prostate cancer surveillance and targeted evaluation of care needs in higher-burden subgroups.

## 1 Introduction

Prostate cancer remains a major cause of cancer morbidity and mortality among men in the United States and worldwide (1, 2). Global projections suggest that its future burden will increase as populations age, including in settings where screening and treatment are well established (3). In the United States, the CDC WONDER provides a national mortality surveillance platform for examining prostate cancer deaths across demographic and geographic subgroups over time (4).

In recent decades, US prostate cancer mortality patterns have reflected the combined influence of PSA-based screening, changes in screening recommendations, treatment advances, and population aging. Published CDC WONDER analyses suggest that the earlier decline in prostate cancer mortality has slowed in recent years, with persistent racial and regional disparities (5, 6). The 2012 USPSTF recommendation against routine PSA-based screening was followed by reduced PSA testing, and later studies have linked this period with less favorable stage patterns and increasing distant-stage disease (7–9). These observations do not establish a single causal pathway but support renewed attention to the burden of advanced and metastatic prostate cancer.

Bone involvement is a central clinical feature of advanced prostate cancer. Prostate cancer is among the solid tumors most strongly associated with bone metastasis, and skeletal dissemination contributes substantially to pain, fractures, spinal cord compression, radiotherapy or surgical needs, and end-of-life morbidity (10–12). Collectively termed skeletal-related events (SREs), these complications, including pathological fractures, spinal cord compression, and the need for palliative radiotherapy or orthopaedic surgery, are a leading cause of intractable bone pain, loss of mobility, impaired quality of life, and substantial health-care costs, underscoring the clinical importance of surveillance focused on bone-metastatic disease (12). Survival rates for distant-stage prostate cancer remain much lower than those for localized disease (13). These clinical consequences are not evenly distributed; studies on prostate cancer outcomes have repeatedly shown worse mortality patterns among Black men and associations with social and neighborhood disadvantages (5, 6, 14, 15).

Most routine mortality summaries rely on the underlying cause of death, which identifies the condition that initiates a fatal sequence. Although useful for standard surveillance, this approach can underestimate clinically relevant conditions recorded on death certificates. CDC WONDER’s Multiple Cause of Death (MCOD) data include both underlying and contributing causes, allowing the identification of deaths in which prostate cancer was the underlying cause and bone metastasis was documented (4, 16). MCOD analysis, which includes deaths where bone metastasis was co-documented as a contributing cause of death, may identify a different mortality trajectory than single underlying cause surveillance.

However, several evidence gaps remain in the literature. Prior US mortality studies have described prostate cancer trends using conventional surveillance approaches; however, national analyses specifically focusing on co-documented prostate cancer and bone metastasis from 1999 to 2024 remain limited (5, 6). Evidence is also sparse on whether this MCOD-defined burden varies by race/ethnicity, region, and urbanization, and how these patterns may evolve over time. Therefore, this study used CDC WONDER MCOD data to examine national mortality trends among deaths with prostate cancer coded as the underlying cause and bone metastasis coded as one of the multiple causes of death between 1999 and 2024. The secondary aim was to evaluate subgroup patterns by age, race/ethnicity, census region, and urbanization, and to generate time-series projections through 2040 to support the planning of advanced prostate cancer care.

## 2 Methods

### 2.1 Study design and setting

This study used a retrospective, population-based surveillance design, applying serial cross-sectional mortality data from 1999 to 2024 to characterize temporal trends and generate conditional projections through 2040. Reporting followed the Strengthening the Reporting of Observational Studies in Epidemiology (STROBE) guidelines (17). The analytical period covered 1999–2024, with time-series projections extending through 2040. The study setting was the United States, which included all 50 states and the District of Columbia (DC).

### 2.2 Data source

Mortality data were obtained from the CDC WONDER Multiple Cause of Death (MCOD) database, which compiles death certificate information submitted by state vital statistics offices to the National Center for Health Statistics (NCHS) (4). Each death certificate includes one underlying cause of death and additional contributing causes, allowing analyses beyond the underlying cause. Population denominators for rate calculation were derived from the intercensal and postcensal estimates embedded within CDC WONDER (4). As the database is publicly available and de-identified, institutional review board approval was not required for this study. As a near-complete census of US death certificates with continuous ICD-10 coding since 1999, the MCOD database constitutes a large, nationally representative sample that supports stable age-adjusted rate estimation and long-term trends and subgroup analyses across the 25-year study period.

### 2.3 Case definition and eligibility

Deaths were included when prostate cancer (ICD-10: C61, malignant neoplasm of the prostate) was listed as the underlying cause of death and bone metastasis (ICD-10: C79.5, secondary malignant neoplasm of the bone and bone marrow) was listed as one of the multiple causes of death. This dual-field approach identifies deaths in which prostate cancer was assigned as the underlying cause and bone metastasis was co-documented on the same death certificate, consistent with the CDC WONDER MCOD methodology (4, 16). Deaths with only one of the two codes were excluded from the study. Specifically, deaths in which bone metastasis (C79.5) was recorded as the underlying cause and deaths in which prostate cancer (C61) appeared only as a contributing cause rather than the underlying cause were not included.

The study population was restricted to male decedents aged ≥25 years. Because prostate cancer is male-specific in this analysis by case definition, no sex or gender stratification was performed. No additional exclusion criteria were applied.

### 2.4 Outcome and stratification variables

The primary outcome was the age-adjusted mortality rate (AAMR) per 100,000 population, standardized to the 2000 US standard population using direct standardization (4). AAMRs were calculated by calendar year and prespecified subgroups.

Stratified analyses were conducted according to age group, race/ethnicity, US Census region, and degree of urbanization. Age groups were classified as 45–64 and 65–85+ years; the 25–44 year group was excluded from trend analysis because of insufficient cell counts for reliable rate estimation (the 25–44 year group contributed only 0–5 deaths per year, remaining below the CDC WONDER suppression threshold of 10 deaths in every year). Race and ethnicity were classified into mutually exclusive categories. Deaths among non-Hispanic White and non-Hispanic Black or African American men were obtained from the CDC WONDER race categories (“White” and “Black or African American,” respectively) with Hispanic origin restricted to “Not Hispanic or Latino,” whereas deaths among Hispanic or Latino men (all races) were queried separately and combined. These non-Hispanic and Hispanic labels were used consistently throughout the manuscript. Cells with fewer than 10 deaths were suppressed according to the CDC WONDER confidentiality standards and excluded from the affected trend analyses (4).

Census regions were classified as Northeast, Midwest, South, or West regions. Urbanization was defined using the 2013 NCHS Urban-Rural Classification Scheme for Counties and was classified into metropolitan and non-metropolitan categories (18). CDC WONDER urbanization data were available until 2020; therefore, projections for this stratum began in 2021. State-level AAMRs and place of death were examined descriptively.

### 2.5 Trend analysis

Joinpoint regression was performed using Joinpoint Trend Analysis Software, version 5.2.0, from the National Cancer Institute (19). Log-linear models were fitted to the annual AAMRs to identify statistically significant changes in trends. The annual percent change (APC), average annual percent change (AAPC), 95% confidence intervals, and p-values were estimated for each age group. A maximum of five joinpoints was allowed per model. This maximum reflects the default configuration of the NCI Joinpoint software for a series of this length and permits the data to support up to five change points; rather than being fixed *a priori*, the final number of joinpoints was selected empirically by the software’s permutation-test procedure, so that additional segments were retained only when they significantly improved the model fit (19). Trends were classified as increasing or decreasing when the APC significantly differed from zero (p < 0.05).

### 2.6 Time-series forecasting

AAMRs were projected from 2025 to 2040 using autoregressive integrated moving average (ARIMA) and error, trend, and seasonal exponential smoothing (ETS) models. These approaches were selected because time-series and model-comparison methods are commonly used in cancer burden forecasting (20–22). The models were fitted in R using the forecast package. The model performance was evaluated using multiple forecast accuracy metrics: mean error, root mean squared error (RMSE), mean absolute error, mean percentage error, mean absolute percentage error (MAPE), mean absolute scaled error, and the lag-1 autocorrelation of residuals, which were computed against a hold-out validation period comprising the most recent years of available data. For each stratum, the model that demonstrated the most favorable overall error profile was retained, and forecasts were reported with 95% prediction intervals.

Model selection was performed separately for the overall series and for each eligible subgroup because mortality trajectories differed across demographic and geographic strata. Sex/gender-specific models were not fitted because all included decedents were male according to the eligibility criteria.

The final model specifications varied across strata, reflecting the differences in the underlying temporal structure of mortality trends. For racial and ethnic groups, ETS(M,A,N) was selected for the non-Hispanic White population, ARIMA(0,2,2) for the Hispanic or Latino population, and ARIMA(0,2,1) for the non-Hispanic Black or African American population, respectively. For age-stratified analyses, ARIMA(1,1,0) with drift was selected for individuals aged 45–64 years, whereas ETS(A,A,N) was selected for those aged 65–85+ years. For the geographic analyses, ETS(A,A,N) was selected for the Northeast, Midwest, and West regions, whereas ARIMA(2,2,1) was selected for the South. For urbanization analyses, ETS(A,A,N) was selected for metropolitan areas and ARIMA(2,2,0) for non-metropolitan areas. For the overall series, ETS(A,A,N) achieved the lowest validation error and was therefore selected for the forecasting.

Among racial and ethnic groups, ARIMA provided a superior fit for the Hispanic or Latino population (RMSE: 0.115; MAPE: 8.39%) and the non-Hispanic Black or African American population (RMSE: 0.187; MAPE: 3.77%), whereas ETS more accurately captured trends in the non-Hispanic White population (RMSE: 0.082; MAPE: 5.66%). Age-stratified analyses similarly revealed heterogeneity in the optimal model type: ARIMA outperformed ETS for the 45–64 age group (RMSE: 0.041; MAPE: 9.20%), whereas ETS was selected for the 65–85+ age group (RMSE: 0.301; MAPE: 5.04%). For the overall series, ETS achieved a marginally lower error profile (RMSE: 0.068; MAPE: 5.07%) and was therefore preferred over the other models.

Geographic stratification followed the same selection criteria as above. Among the four US Census Regions, the ETS was the better-fitting model for the Northeast (RMSE: 0.077; MAPE: 7.60%), Midwest (RMSE: 0.110; MAPE: 8.39%), and West (RMSE: 0.120; MAPE: 6.88%), whereas ARIMA achieved greater accuracy in the South (RMSE: 0.065; MAPE: 4.21%). Analyses stratified by urbanization status yielded a similar pattern: ETS was selected for metropolitan areas (RMSE: 0.066; MAPE: 5.43%), and ARIMA for non-metropolitan regions (RMSE: 0.073; MAPE: 4.99%).

Projections were extended to 2040 to estimate the potential future burden of disease and inform long-term public health planning. Forecasts are presented with 95% prediction intervals, which widen progressively over the forecast horizon, reflecting increasing uncertainty in long-term projections. Accordingly, the projected values should be interpreted as estimates of potential future trends rather than precise predictions.

### 2.7 Sensitivity analyses

Two sensitivity analyses were conducted. First, the analysis was repeated using an underlying cause-only definition for prostate cancer without requiring bone metastasis co-documentation for prostate cancer. This allowed a comparison between conventional underlying cause surveillance and the MCOD dual-field approach (4, 16).

Second, a pre-pandemic sensitivity analysis was conducted by restricting the trend model to the 1999–2019 data. This assessed whether the observed pre-2020 trend pattern was consistent before the major COVID-era disruptions to cancer screening and diagnosis (23, 24).

### 2.8 Descriptive analysis

State-level AAMRs were examined using percentile ranking to identify states with the highest and lowest mortality burdens. The place of death was summarized using the proportions across the CDC WONDER categories.

## 3 Results

### 3.1 Overall mortality trends and projections

**Figure 1:**
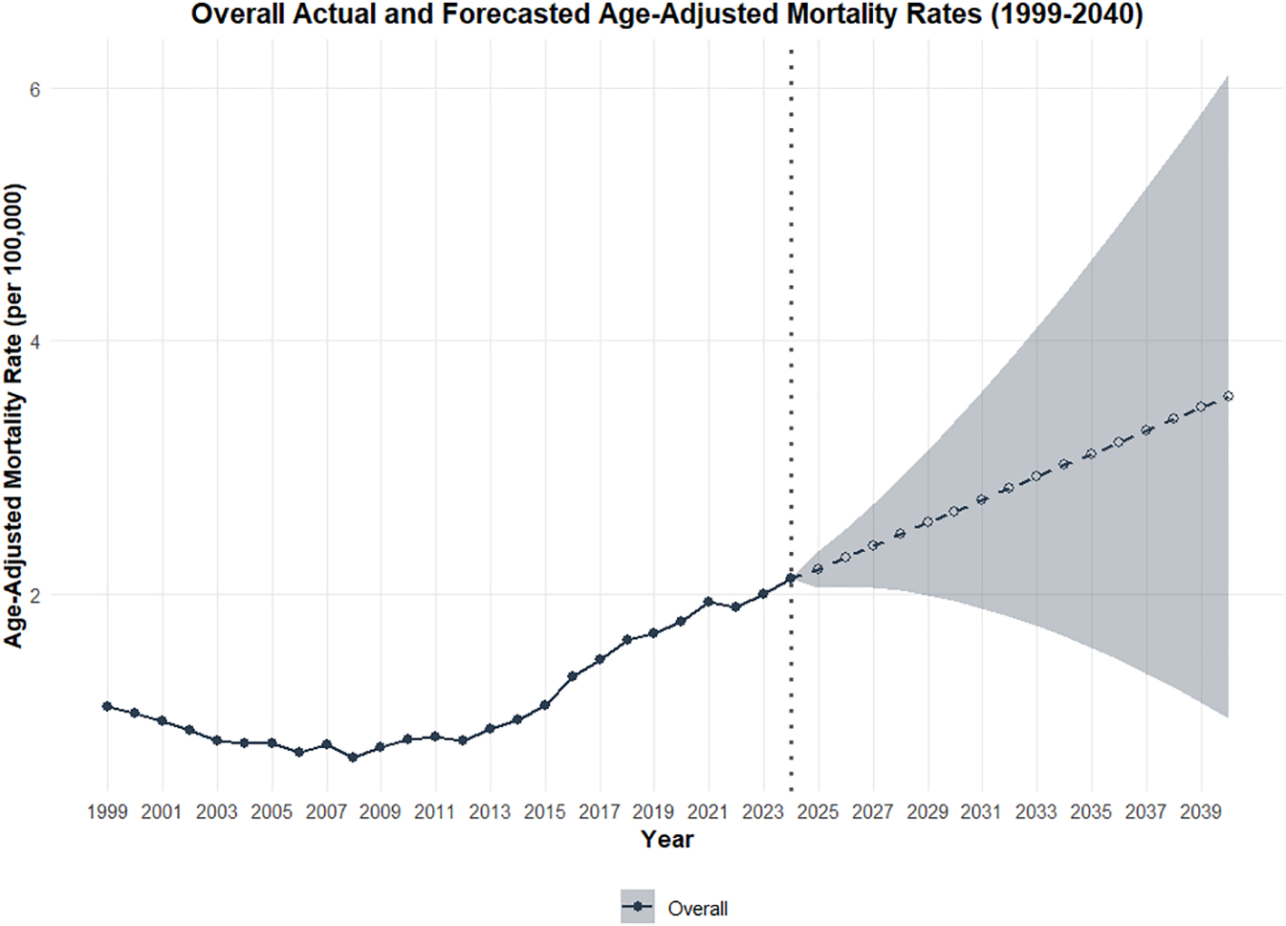
Overall age-adjusted mortality rates for prostate cancer deaths with co-documented bone metastasis: observed trends (1999–2024) and time-series forecasts (2025–2040) in the United States.

Between 1999 and 2024, 73,429 deaths were recorded with prostate cancer (ICD-10: C61) as the underlying cause and bone metastasis (ICD-10: C79.5) as a contributing cause, yielding a mean AAMR of 1.19 per 100,000 over the surveillance period (**Table 1**; **Figure 1**; **Supplementary Table 1**). The AAMR rose from 1.10 (95% CI: 1.05–1.15) in 1999 to 2.12 (95% CI: 2.06–2.17) in 2024, representing a 93% increase over 25 years (AAPC: +2.61; 95% CI: 1.66–3.56; p < 0.000001).

**Table 1:** Annual percent change and average annual percent change in age-adjusted mortality rates, 1999-2024.

Category	Cohort	Year segment	APC, % (95% CI)	APC p-value	AAPC, % (95% CI)	AAPC p-value
Overall	Overall male-only cohort	1999–2006	–5.57 (–7.01, –4.11)	0.000001	2.61 (1.66, 3.56)	<0.000001
		2006–2013	2.92 (0.63, 5.26)	0.015572		
		2013–2018	12.91 (9.77, 16.14)	<0.000001		
		2018–2024	4.02 (2.53, 5.54)	0.000034		
Urbanization	Metropolitan	1999–2003	–7.20 (–10.44, –3.85)	0.001264	2.25 (0.63, 3.90)	0.006298
		2003–2008	–1.96 (–5.35, 1.55)	0.230082		
		2008–2014	4.52 (2.06, 7.04)	0.002690		
		2014–2017	16.98 (7.08, 27.80)	0.003479		
		2017–2020	4.42 (0.42, 8.57)	0.033956		
Urbanization	Non-metropolitan	1999–2009	–4.79 (–5.91, –3.65)	<0.000001	2.31 (1.63, 2.99)	<0.000001
		2009–2020	9.21 (8.29, 10.14)	<0.000001		
Age group	45–64 years	1999–2008	–1.74 (–5.06, 1.69)	0.299022	3.28 (1.92, 4.65)	0.000002
		2008–2024	6.21 (5.13, 7.31)	<0.000001		
Age group	65–85+ years	1999–2008	–4.59 (–5.59, –3.58)	<0.000001	2.73 (1.47, 4.00)	0.000019
		2008–2014	5.49 (2.79, 8.25)	0.000513		
		2014–2017	16.26 (6.08, 27.41)	0.003195		
		2017–2024	4.73 (3.81, 5.65)	<0.000001		
Race/ethnicity	Hispanic or Latino	1999–2008	–5.80 (–8.82, –2.67)	0.001187	1.53 (–0.04, 3.13)	0.056321
		2008–2019	8.12 (5.93, 10.35)	<0.000001		
		2019–2024	1.20 (–2.71, 5.26)	0.532564		
Race/ethnicity	Non-Hispanic Black or African American	1999–2004	–9.96 (–14.04, –5.69)	0.000347	1.11 (–1.27, 3.54)	0.365666
		2004–2014	0.05 (–2.01, 2.15)	0.958190		
		2014–2018	14.79 (5.30, 25.14)	0.004531		
		2018–2021	–0.02 (–13.35, 15.37)	0.998196		
		2021–2024	8.44 (1.66, 15.66)	0.018101		
Race/ethnicity	Non-Hispanic White	1999–2008	–4.00 (–5.35, –2.62)	0.000020	3.16 (1.55, 4.80)	0.000108
		2008–2014	5.65 (2.42, 8.98)	0.001834		
		2014–2017	17.58 (4.50, 32.29)	0.010405		
		2017–2024	4.82 (3.54, 6.12)	0.000001		
Census region	Northeast	1999–2012	–4.84 (–6.16, –3.50)	<0.000001	1.07 (0.16, 1.99)	0.020436
		2012–2024	7.89 (6.50, 9.29)	<0.000001		
Census region	Midwest	1999–2012	–3.98 (–5.17, –2.77)	0.000002	2.62 (1.28, 3.98)	0.000115
		2012–2018	15.49 (10.28, 20.94)	0.000004		
		2018–2024	5.33 (2.79, 7.93)	0.000299		
Census region	South	1999–2006	–6.52 (–8.29, –4.72)	0.000002	2.34 (0.76, 3.94)	0.003557
		2006–2014	4.57 (2.38, 6.81)	0.000427		
		2014–2017	15.32 (2.43, 29.84)	0.021593		
		2017–2024	3.86 (2.74, 5.01)	0.000002		
Census region	West	1999–2006	–1.74 (–5.35, 2.00)	0.332103	4.75 (2.86, 6.67)	0.000001
		2006–2014	7.30 (4.09, 10.60)	0.000175		
		2014–2018	14.97 (6.10, 24.58)	0.002132		
		2018–2024	2.72 (0.68, 4.80)	0.012135		

AAPC, average annual percent change; APC, annual percent change; CI, confidence interval. APC and AAPC estimates were derived from the Joinpoint regression. Urbanization estimates use 1999–2020 data because urban classification data were available through 2020, and all other categories use 1999–2024 data. The P values correspond to the APC or AAPC estimates shown in the same row.

Joinpoint regression identified three inflection points, producing four distinct trend segments (**Figure 1**). AAMRs declined significantly from 1999 to 2006 (APC: −5.57; 95% CI: −7.01 to −4.11; p = 0.000001), reaching a nadir of 0.74 per 100,000 in 2006. A modest but significant increase was observed from 2006 to 2013 (APC: +2.92, 95% CI: 0.63–5.26, p = 0.016). The rate of increase accelerated sharply from 2013 to 2018 (APC: +12.91; 95% CI: 9.77–16.14; p < 0.000001) and continued at a more moderate but sustained pace from 2018 to 2024 (APC: +4.02; 95% CI: 2.53–5.54; p < 0.001).

Time-series forecasting projected the AAMR to rise from 2.29 (95% CI: 2.06–2.52) in 2025 to 3.65 (95% CI: 0.88–6.43) per 100,000 by 2040, representing a further 59% increase relative to the projected 2025 value (equivalent to a 72% increase above the 2024 observed rate of 2.12/100,000). The 95% prediction interval widens markedly by 2040 (0.88–6.43), indicating considerable forecast uncertainty (**Supplementary Table 2**; **Figure 1**).

The observed annual AAMRs per 100,000 population are plotted as individual data points. Joinpoint regression segments are shown with their estimated annual percent changes (APCs). The dashed vertical line separates the observed and projected values. The shaded bands indicate the 95% prediction intervals for the ETS forecast. AAMR, age-adjusted mortality rate; APC, annual percent change; ETS, error, trend, and seasonal exponential smoothing model.

### 3.2 Age-stratified trends and projections

A total of 176 deaths were recorded in the 45–64 age group in 1999, which rose to 565 deaths by 2024. Among those aged 65–85+, deaths increased from 1,771 to 5,513 during the same period (**Supplementary Table 3**). The mortality burden was heavily concentrated in older men during the study period.

**Figure 2:**
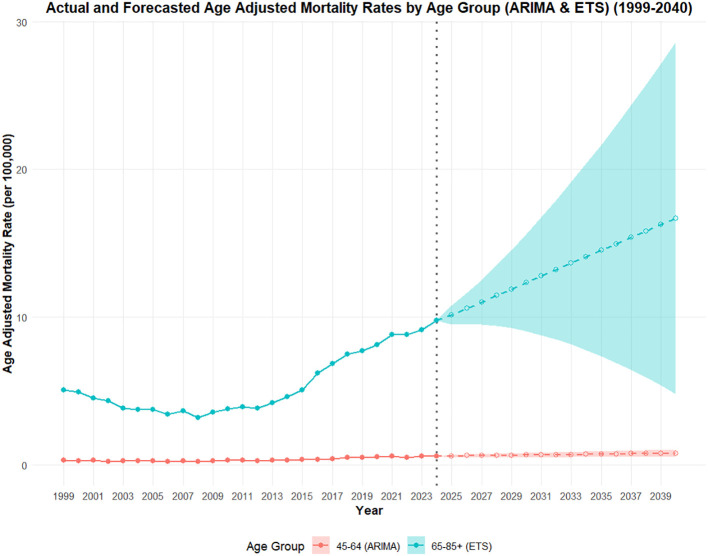
Age-adjusted mortality rates for prostate cancer deaths with co-documented bone metastasis by age group: observed trends (1999–2024) and time-series forecasts (2025–2040) in the United States.

In the 45–64-year age group, the AAMR rose from 0.30 (95% CI: 0.25–0.34) in 1999 to 0.59 (95% CI: 0.55–0.64) in 2024 (AAPC: +3.28; 95% CI: 1.92–4.65; p = 0.000002). Joinpoint analysis identified one inflection point in 2008 (95% CI: 2004–2011), producing two trend segments. AAMRs declined non-significantly from 1999 to 2008 (APC: −1.74; 95% CI: −5.06 to 1.69; p = 0.299), followed by a sustained and significant rise from 2008 to 2024 (APC: +6.21; 95% CI: 5.13–7.31; p < 0.000001) (**Table 1**; **Figure 2**; **Supplementary Table 3**).

In the 65–85+ year age group, the AAMR rose from 5.09 (95% CI: 4.86–5.33) in 1999 to 9.79 (95% CI: 9.53–10.05) in 2024 (AAPC: +2.73; 95% CI: 1.47–4.00; p = 0.000019). Joinpoint analysis identified three inflection points in 2008 (95% CI: 2003–2009), 2014 (95% CI: 2011–2016), and 2017 (95% CI: 2016–2020), producing four trend segments. AAMRs declined significantly from 1999 to 2008 (APC: −4.59; 95% CI: −5.59 to −3.58; p < 0.000001). A significant rise followed from 2008 to 2014 (APC: +5.49; 95% CI: 2.79–8.25; p = 0.000513), succeeded by a sharp acceleration from 2014 to 2017 (APC: +16.26; 95% CI: 6.08–27.41; p = 0.003195). The rates continued to increase significantly from 2017 to 2024 (APC: +4.73, 95% CI: 3.81–5.65, p < 0.000001).

Projections through 2040 show a divergence between the age groups (**Supplementary Table 4**; **Figure 2**). Among those aged 45–64 years, the AAMR rose gradually from 0.62 (95% CI: 0.52–0.71) in 2025 to 0.81 (95% CI: 0.56–1.05) in 2040, a 31% increase. Among those aged 65–85+, the AAMR rose from 10.60 (95% CI: 9.56–11.63) in 2025 to 17.14 (95% CI: 4.19–30.09) in 2040, a 62% projected increase, although the wide prediction interval (4.19–30.09) reflects considerable uncertainty in the estimate.

Separate panels display results for the 45–64 and 65–85+ age groups. The dashed vertical line separates the observed and projected values. The shaded bands indicate the 95% prediction intervals. AAMR, age-adjusted mortality rate; APC, annual percent change; ARIMA, autoregressive integrated moving average; ETS, error, trend, and seasonal exponential smoothing model.

### 3.3 Racial and ethnic disparities in trends and projections

Across the full surveillance period, non-Hispanic Black or African American men had the highest mean AAMR at 1.89 per 100,000, with 10,417 deaths, compared with 1.15 per 100,000 among non-Hispanic White men, with 56,125 deaths, and 1.07 per 100,000 among Hispanic or Latino men, with 5,088 deaths. This corresponds to an approximately 64% higher mean AAMR among non-Hispanic Black or African American men than among non-Hispanic White men across the study period (**Supplementary Table 5**).

Among Hispanic or Latino men, the AAMR increased from 1.23 (95% CI: 0.98–1.48) in 1999 to 1.62 (95% CI: 1.47–1.78) by 2024. The overall AAPC was +1.53% (95% CI: −0.04 to 3.13; p = 0.056), which was not statistically significant. Joinpoint analysis showed a significant decline from 1999 to 2008 (APC: −5.80%; 95% CI: −8.82 to −2.67; p = 0.001), followed by a significant increase from 2008 to 2019 (APC: +8.12%; 95% CI: 5.93 to 10.35; p < 0.000001) and a non-significant increase from 2019 to 2024.

Among non-Hispanic Black or African American men, the AAMR increased from 2.47 (95% CI: 2.21–2.73) in 1999 to 3.09 (95% CI: 2.89–3.31) by 2024. The overall AAPC was +1.11% (95% CI: −1.27 to 3.54; p = 0.366), which was not significant. Joinpoint analysis showed a significant decline from 1999 to 2004 (APC: −9.96%; 95% CI: −14.04 to −5.69; p < 0.001), no significant change from 2004 to 2014, a significant increase from 2014 to 2018 (APC: +14.79%; 95% CI: 5.30 to 25.14; p = 0.005), no significant change from 2018 to 2021, and a renewed significant increase from 2021 to 2024 (APC: +8.44%; 95% CI: 1.66 to 15.66; p = 0.018).

**Figure 3:**
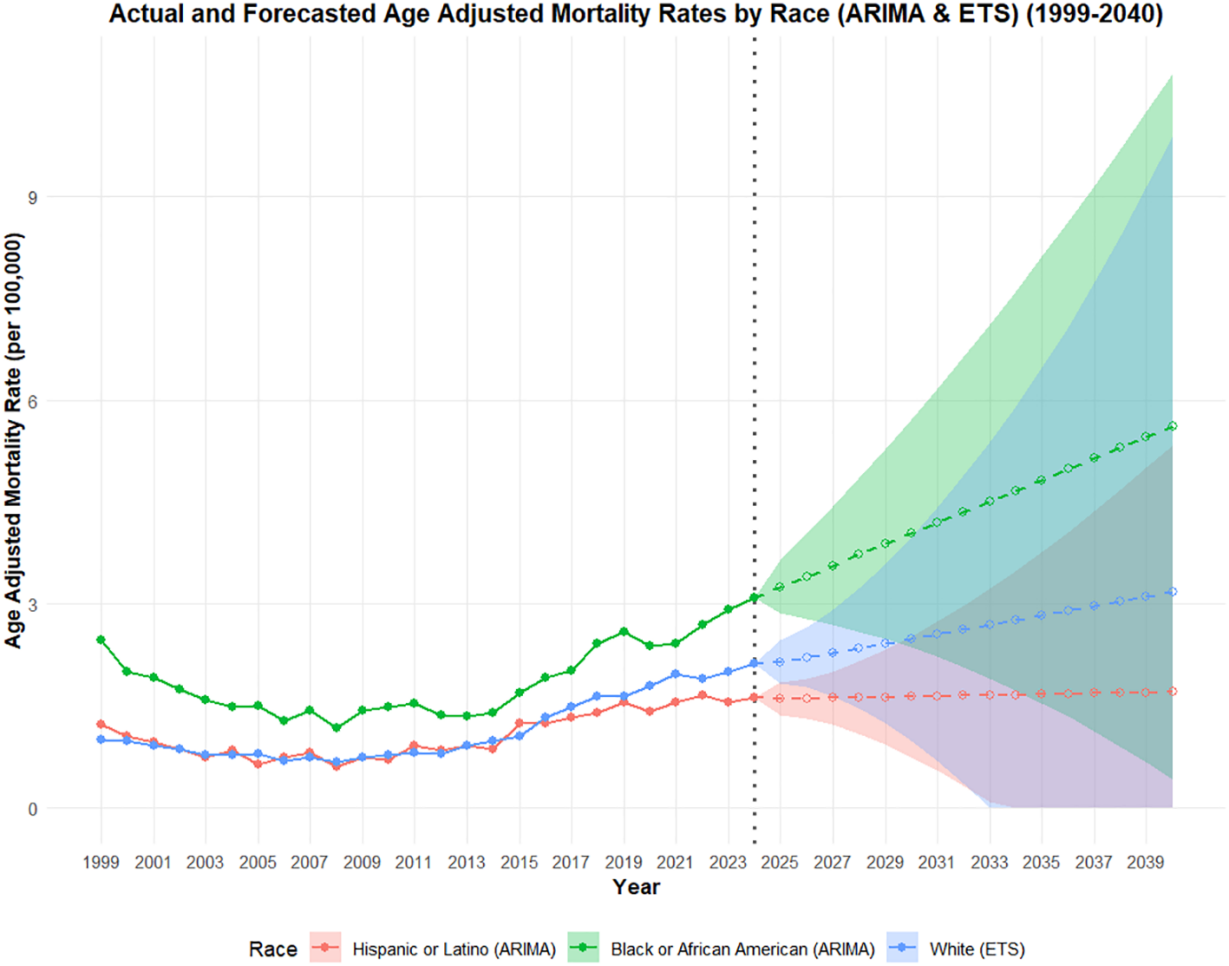
Age-adjusted mortality rates for prostate cancer deaths with co-documented bone metastasis by race and ethnicity: observed trends (1999–2024) and time-series forecasts (2025–2040) in the United States.

Among non-Hispanic White men, the AAMR increased from 1.00 (95% CI: 0.95–1.05) in 1999 to 2.12 (95% CI: 2.06–2.18) in 2024. The overall AAPC was +3.16% (95% CI: 1.55 to 4.80; p < 0.001), the largest statistically significant overall increase among the three groups. Joinpoint analysis showed a significant decline from 1999 to 2008 (APC: −4.00%; 95% CI: −5.35 to −2.62; p < 0.001), followed by significant increases from 2008 to 2014 (APC: +5.65%; 95% CI: 2.42 to 8.98; p = 0.002), from 2014 to 2017 (APC: +17.58%; 95% CI: 4.50 to 32.29; p = 0.010), and from 2017 to 2024 (APC: +4.82%; 95% CI: 3.54 to 6.12; p < 0.000001). (**Table 1**; **Figure 3**; **Supplementary Table 5**).

Model-based projections through 2040 suggest that racial differences may widen in absolute terms if recent trends continue (**Supplementary Table 6**; **Figure 3**). Among non-Hispanic Black or African American men, the projected AAMR increased from 3.41 (95% CI: 2.77–4.04) in 2025 to 5.77 (95% CI: 0.16–11.38) in 2040, a projected increase of approximately 69%. Among non-Hispanic White men, the projected AAMR increased from 2.21 (95% CI: 1.77–2.66) in 2025 to 3.24 (95% CI: 0.00–10.71) in 2040, representing a projected increase of approximately 47%. Hispanic or Latino men showed a smaller projected increase, from 1.61 (95% CI: 1.32–1.91) in 2025 to 1.71 (95% CI: 0.00–5.69) by 2040. The projected ratio of non-Hispanic Black or African American to non-Hispanic White AAMRs increased from 1.54 in 2025 to 1.78 in 2040, suggesting a widening relative disparity under the projection model. (**Supplementary Table 6**).

Trend lines are displayed separately for non-Hispanic Black or African American, non-Hispanic White, and Hispanic or Latino male decedents. In the figure legend, “Black or African American” and “White” denote the corresponding, non-Hispanic groups. The dashed vertical line separates the observed and projected values. The shaded bands indicate the 95% prediction intervals. AAMR, age-adjusted mortality rate; ARIMA, autoregressive integrated moving average; ETS, error, trend, and seasonal exponential smoothing model.

### 3.4 Geographic variation by census region

The South recorded the highest absolute death toll over the surveillance period (28,817 deaths), followed by the West (18,926 deaths), Midwest (16,594 deaths), and Northeast (9,092 deaths). In terms of the mean AAMR, however, the West carried the highest burden over the study period at 1.38 per 100,000, followed by the South (1.27), Midwest (1.22), and Northeast (0.78) (**Supplementary Table 7**).

In the Northeast, the AAMR increased from 1.04 (95% CI: 0.94–1.14) in 1999 to 1.27 (95% CI: 1.17–1.37) in 2024 (AAPC: +1.07; 95% CI: 0.16–1.99; p = 0.020). A significant decline occurred from 1999 to 2012 (APC: −4.84; 95% CI: −6.16 to −3.50; p < 0.000001), followed by a significant reversal from 2012 to 2024 (APC: +7.89; 95% CI: 6.50–9.29; p < 0.000001).

In the Midwest, the AAMR increased from 1.16 (95% CI: 1.06–1.26) in 1999 to 2.29 (95% CI: 2.17–2.42) in 2024 (AAPC: +2.62; 95% CI: 1.28–3.98; p < 0.001). After a significant early decline until 2012 (APC: −3.98; 95% CI: −5.17 to −2.77; p < 0.001), a sharp increase occurred from 2012 to 2018 (APC: +15.49; 95% CI: 10.28–20.94; p < 0.001), followed by a continued significant growth from 2018 to 2024 (APC: +5.33; 95% CI: 2.79–7.93; p < 0.001).

In the South, the AAMR increased from 1.24 (95% CI: 1.15–1.33) in 1999 to 2.15 (95% CI: 2.06–2.24) in 2024 (AAPC: +2.34; 95% CI: 0.76–3.94; p = 0.004). A significant early decline extended through 2006 (APC: −6.52; 95% CI: −8.29 to −4.72; p < 0.001), followed by a significant rise from 2006 to 2014 (APC: +4.57; 95% CI: 2.38–6.81; p < 0.001), acceleration from 2014 to 2017 (APC: +15.32; 95% CI: 2.43–29.84; p = 0.022), and continued increase from 2017 to 2024 (APC: +3.86; 95% CI: 2.74–5.01; p < 0.001).

**Figure 4:**
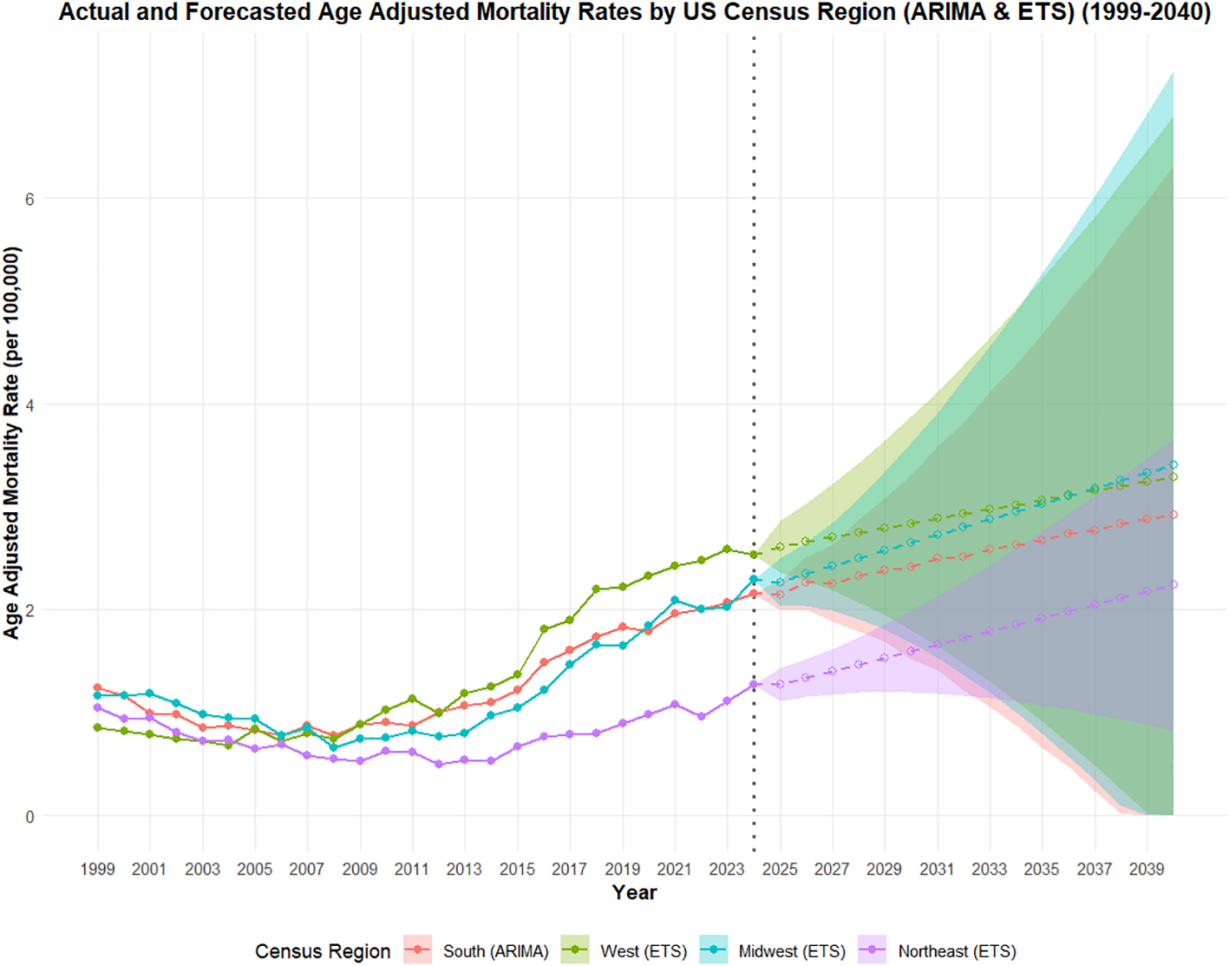
Age-adjusted mortality rates for prostate cancer deaths with co-documented bone metastasis by US Census region: observed trends (1999–2024) and time-series forecasts (2025–2040), United States.

In the West, the AAMR increased from 0.85 (95% CI: 0.75–0.95) in 1999 to 2.53 (95% CI: 2.41–2.66) in 2024, a 198% increase, yielding the highest AAPC of any region (+4.75; 95% CI: 2.86–6.67; p < 0.000001). After a non-significant initial decline until 2006 (APC: −1.74; p = 0.332), significant increases were recorded from 2006 to 2014 (APC: +7.30; p < 0.001), 2014 to 2018 (APC: +14.97; p = 0.002), and 2018 to 2024 (APC: +2.72; p = 0.012). (**Table 1**; **Figure 4**; **Supplementary Table 7**).

Projections through 2040 show rising AAMRs across all four regions, with the West maintaining the highest rate (**Supplementary Table 8**; **Figure 4**). The West is projected to reach 3.33 (95% CI: 0.00–7.15) per 100,000 by 2040 from 2.66 (95% CI: 2.28–3.03) in 2025. The Midwest rises from 2.34 (95% CI: 2.03–2.65) to 3.48 (95% CI: 0.00–7.67). The South increases from 2.26 (95% CI: 2.01–2.51) to 2.98 (95% CI: 0.00–6.68). The Northeast had the lowest rate, which increased from 1.33 (95% CI: 1.15–1.51) to 2.30 (95% CI: 0.76–3.84). (**Supplementary Table 8**).

Trend lines are displayed separately for the Northeast, Midwest, South, and West regions. The dashed vertical line separates the observed and projected values. The shaded bands indicate the 95% prediction intervals. AAMR, age-adjusted mortality rate; APC, annual percent change; ARIMA, autoregressive integrated moving average; ETS, error, trend, and seasonal exponential smoothing model.

### 3.5 Urbanization trends and projections

**Figure 5:**
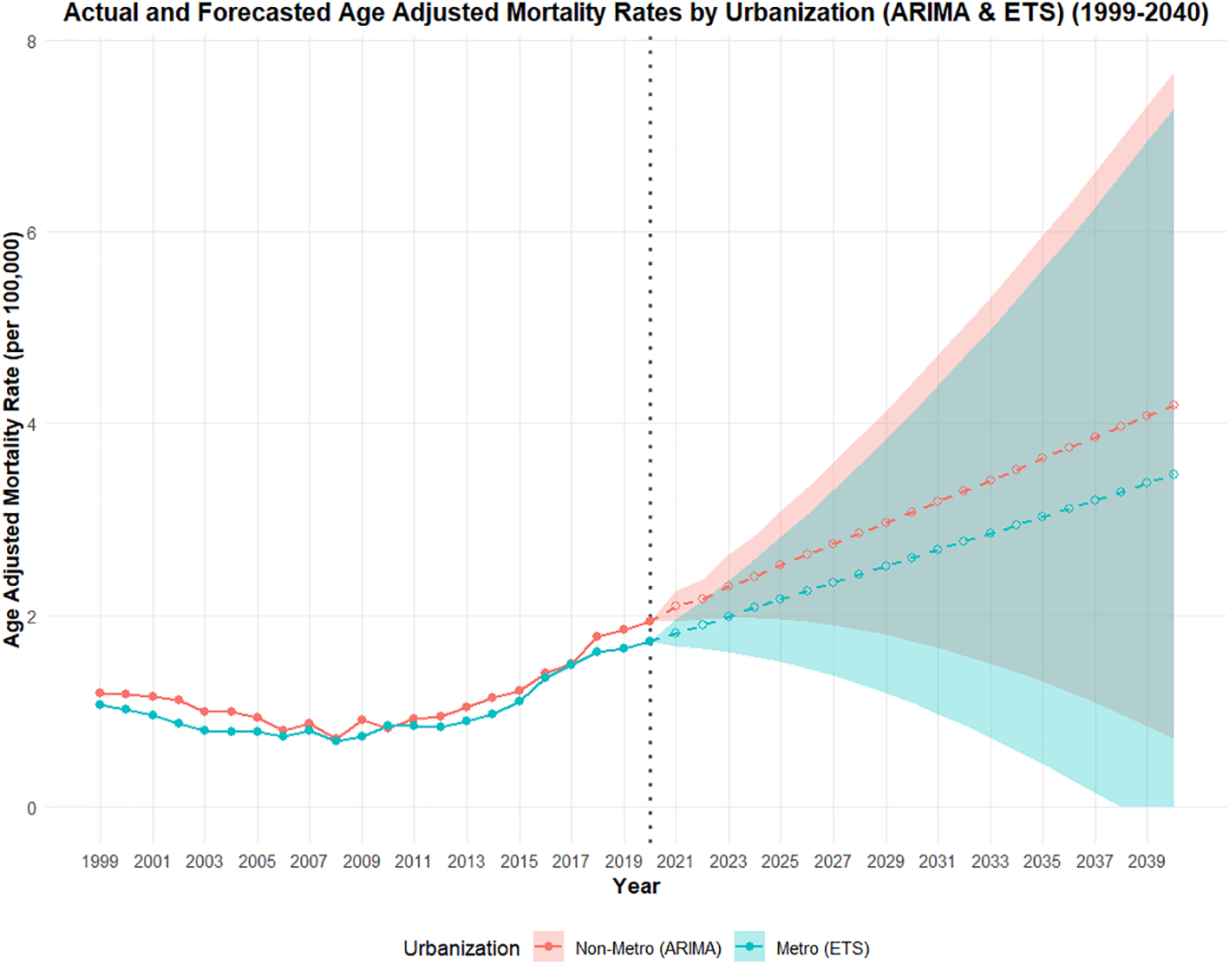
Age-adjusted mortality rates for prostate cancer deaths with co-documented bone metastasis by urbanization status: observed trends (1999–2020) and time-series forecasts (2021–2040) in the United States.

Urbanization data were available from 1999 to 2020 in the CDC WONDER database. Non-metropolitan areas recorded a higher mean AAMR of 1.15 per 100,000 (9,987 deaths) than metropolitan areas (1.02 per 100, 000; 41,470 deaths) during this period (**Table 1**; **Figure 5**; **Supplementary Table 9**).

In metropolitan areas, the AAMR increased from 1.07 (95% CI: 1.02–1.13) in 1999 to 1.73 (95% CI: 1.68–1.79) in 2020 (AAPC: +2.25; 95% CI: 0.63–3.90; p = 0.006). Joinpoint analysis identified a significant early decline from 1999 to 2003 (APC: −7.20; 95% CI: −10.44 to −3.85; p = 0.001), a non-significant further decline from 2003 to 2008, followed by a significant rise from 2008 to 2014 (APC: +4.52; 95% CI: 2.06–7.04; p = 0.003), a sharp acceleration from 2014 to 2017 (APC: +16.98; 95% CI: 7.08–27.80; p = 0.004), and a continued moderate increase from 2017 to 2020 (APC: +4.42; 95% CI: 0.42–8.57; p = 0.034).

In non-metropolitan areas, the AAMR increased from 1.19 (95% CI: 1.08–1.31) in 1999 to 1.94 (95% CI: 1.81–2.07) in 2020 (AAPC: +2.31; 95% CI: 1.63–2.99; p < 0.000001). A single joinpoint model identified a significant early decline from 1999 to 2009 (APC: −4.79; 95% CI: −5.91 to −3.65; p < 0.000001), followed by a sharp and sustained increase from 2009 to 2020 (APC: +9.21; 95% CI: 8.29–10.14; p < 0.000001).

Projections from 2021 to 2040 show that non-metropolitan rates consistently exceed metropolitan rates and diverge over time (**Supplementary Table 10**; **Figure 5**). Metropolitan AAMRs are projected to rise from 1.82 (95% CI: 1.67–1.96) in 2021 to 3.46 (95% CI: 0.00–7.29) by 2040. Non-metropolitan rates are projected to rise from 2.09 (95% CI: 1.94–2.25) in 2021 to 4.19 (95% CI: 0.71–7.67) in 2040, a 100% increase from the 2021 baseline, indicating that the rural-urban mortality gap is projected to persist and widen by 2040. (**Supplementary Table 10**).

Trend lines are displayed separately for metropolitan and non-metropolitan areas. Urbanization-stratified surveillance data in CDC WONDER are available only until 2020; therefore, projections commence from 2021. The dashed vertical line separates the observed and projected values. The shaded bands indicate the 95% prediction intervals. AAMR, age-adjusted mortality rate; ARIMA, autoregressive integrated moving average; ETS, error, trend, and seasonal exponential smoothing model.

### 3.6 State-level distribution

From 1999 to 2020, Nevada had the highest average state-level AAMR at 2.16 per 100,000, whereas Massachusetts had the lowest at 0.46 per 100,000. The states in the highest 10% of AAMRs were Nevada, Arkansas, Mississippi, Vermont, Texas, and California, whereas those in the lowest 10% were Massachusetts, Connecticut, Rhode Island, the District of Columbia, and Maryland.

From 2021 to 2024, Oregon had the highest state-level AAMR at 4.86 per 100,000, whereas New Jersey had the lowest at 0.60 per 100,000. The states with the highest burden of AAMR were Oregon, Nevada, Vermont, Hawaii, and Idaho, whereas those with the lowest burden were New Jersey, Rhode Island, Louisiana, the District of Columbia, and Massachusetts (**Supplementary Table 11**). These state-level estimates should be interpreted with caution because small case counts and CDC WONDER cell-suppression rules can destabilize age-adjusted rates in less populous states.

### 3.7 Place of death

Place-of-death data were available for 51,457 deaths between 1999 and 2020. Of these, the majority (24,962, 48.51%) occurred in the decedent’s home (**Supplementary Table 12**). Nursing homes or long-term care facilities accounted for 9,496 deaths (18.45%), followed by inpatient medical (8,185 deaths; 15.91%) and hospice (5, 386 deaths; 10.47%) facilities. Other locations accounted for 2,698 deaths (5.24%), outpatient or emergency settings for 594 deaths (1.15%), deaths classified as dead on arrival for 42 deaths (0.08%), status unknown for 27 deaths (0.05%), and place of death unknown for 67 deaths (0.13%).

### 3.8 Sensitivity analyses

Pandemic exclusion: When the analysis was restricted to 1999–2019, excluding the pandemic years 2020–2024, Joinpoint software identified two inflection points at 2006 and 2012, producing three trend segments: a significant decline from 1999 to 2006 (APC: −5.42; 95% CI: −7.18 to −3.62; p < 0.001), a non-significant rise from 2006 to 2012 (APC: +2.25; 95% CI: −1.35 to 5.99; p = 0.203), and a sharp significant acceleration from 2012 to 2019 (APC: +11.07; 95% CI: 9.21–12.95; p < 0.000001). The overall AAPC for 1999–2019 was +2.42 (95% CI: 1.14–3.72; p < 0.001). These estimates are consistent with those from the full 1999–2024 model (AAPC: +2.61; post-2013 APC: +12.91), confirming that the acceleration in bone-metastatic prostate cancer mortality predates the pandemic and that the inclusion of 2020–2024 data does not materially alter the direction or magnitude of the trend estimates.

Underlying cause only: When the analysis was restricted to deaths with prostate cancer (C61) as the sole underlying cause of death without requiring bone metastasis documentation in the multiple cause field, Joinpoint identified a single inflection in 2013, yielding a sustained and significant decline from 1999 to 2013 (APC: −2.80; 95% CI: −2.97 to −2.63; p < 0.000001), followed by a non-significant plateau from 2013 to 2024 (APC: +0.06; 95% CI: −0.19 to 0.31; p = 0.611). The AAPC for the full period was −1.55 (95% CI: −1.69 to −1.41; p < 0.000001), which was directionally opposite to that of the primary MCOD dual-field model. This divergence suggests that the rising AAMR trajectory in the primary analysis is primarily captured by the concurrent documentation of bone metastasis as a contributing cause, a signal that is entirely absent from conventional single-cause analyses. This contrast indicates that co-documented bone metastasis in the MCOD framework captures a component of prostate cancer mortality that does not appear in underlying-cause-only summaries, although whether this reflects true metastatic burden or documentation variation cannot be determined from death certificate data alone (**Supplementary Table 13**).

## 4 Discussion

### 4.1 Principal findings and temporal mortality patterns

This national surveillance analysis identified 73,429 deaths from 1999 to 2024, in which prostate cancer was recorded as the underlying cause of death and bone metastasis was co-documented as one of the multiple causes of death. The overall AAMR increased from 1.10 to 2.12 per 100,000 during the study period, whereas the conventional underlying cause-only comparison showed a different temporal pattern of change. This contrast suggests that underlying cause-only surveillance may underrepresent the contribution of co-documented osseous metastatic disease to prostate cancer mortality (4–6, 16).

The MCOD approach is useful in this context because bone-metastatic prostate cancer can contribute to the terminal course through several complications, including pathological fractures, spinal cord compression, immobility-related complications, and palliative procedural needs (11, 12, 16). However, the interpretation of these results should be approached cautiously. MCOD data depend on death certificate documentation, and the accuracy of prostate cancer death certificates is imperfect, particularly in clinically complex cases or when another malignancy is present (25). Therefore, the observed increase should be interpreted as an increase in documented prostate cancer deaths with co-recorded bone metastasis, not as direct proof of a biological increase in the metastatic burden alone.

The joinpoint pattern showed an early decline, followed by an increase. The post-2013 increase occurred in the same period as the years following the 2012 USPSTF recommendation against routine PSA-based screening; separately, studies have documented reduced PSA testing and later-stage presentation patterns during that era (7–9). This study did not establish a connection between these observations. The ecological design precludes causal interpretation, and the co-occurrence of these patterns supports closer monitoring of advanced-stage and bone-metastatic prostate cancer outcomes rather than any inference about the mechanism.

The 2013–2018 acceleration (APC + 12.91%) merits particular attention because it was the steepest segment in the series and coincided with several concurrent developments that could not be disentangled using death certificate data. First, independent registry data point to a genuine rise in advanced disease: analyses of the SEER and the National Cancer Database have documented an increase in the incidence of distant-stage and metastatic prostate cancer of roughly 4–7% per year beginning around 2010–2011, in the period following the 2012 USPSTF recommendation against routine PSA-based screening and the associated decline in PSA testing (7–9). The timing and direction of the present MCOD mortality signal are therefore concordant with these incidence trends, suggesting that at least part of the increase reflects a true growth in the bone-metastatic disease burden. Second, the same interval saw wider use of cross-sectional and advanced imaging, greater clinical recognition of metastatic disease, and more detailed end-of-life and death certificate documentation, each of which could increase the probability that bone metastasis is recorded as a contributing cause, independently of any change in true incidence (16, 25). Third, secular changes in multiple-cause coding practices may also contribute to this (16). These mechanisms are not mutually exclusive, and the present data cannot apportion the observed acceleration among them; however, concordance with external incidence data argues against documentation change as the sole explanation.

The slower post-2018 increase also requires cautious interpretation. CDC WONDER does not include treatment exposure, disease stage at diagnosis, imaging history, or comorbidity data of patients. Consequently, the observed post-2018 pattern cannot be attributed to treatment uptake, pandemic-related disruption, documentation changes, or any other explanation. Second-generation androgen receptor pathway inhibitors have shown survival benefits in metastatic hormone-sensitive prostate cancer trials (26–29); however, whether their uptake affected the population-level mortality trajectory observed here cannot be assessed from death certificate data alone.

The projections for 2040 should be treated as conditional planning estimates, rather than fixed predictions. The use of ARIMA, ETS, RMSE, and MAPE is consistent with the published approaches to cancer burden forecasting (20–22). However, the wide prediction intervals indicate substantial long-term uncertainties. These projections are best used to frame potential future service needs, not to make precise claims about future mortality.

### 4.2 Divergence between MCOD and underlying-cause surveillance

The comparison between the MCOD dual-field definition and the underlying cause-only definition was a central methodological observation. The underlying cause-only model was consistent with published reports showing declining or slowing prostate cancer mortality trends in conventional surveillance systems (5, 6). In contrast, the MCOD-defined series increased during this period. This suggests that co-documented bone metastasis captures a clinically relevant subset of prostate cancer mortality that is not visible in underlying cause-only summaries (4, 16).

Several explanations are plausible and cannot be separated using the design of this study. First, the rising incidence of coded distant-stage prostate cancer observed in surveillance data concurrent with changes in screening patterns may have increased the proportion of deaths with bone metastasis co-documented on death certificates (7–9). Second, improvements in imaging and staging, including the broader use of PSMA PET/CT, may have increased the recognition and documentation of bone metastases on death certificates (30, 31) because PSMA PET/CT was not widely adopted in US practice until after its initial FDA approvals in 2020–2021; however, this mechanism is more likely to have affected documentation in the most recent years than the 2013–2018 acceleration. Third, documentation practices may have changed over time, independently of the disease burden (16, 25). These explanations are not mutually exclusive or exhaustive.

The pandemic period raised another interpretive issue. Hansen et al. reported that COVID-19 was often assigned as the underlying cause when cancer was listed as a contributing condition during the pandemic period (32). However, the present analysis required prostate cancer to be the underlying cause of death. Therefore, deaths in which COVID-19 was assigned as the underlying cause and prostate cancer was only contributory would not be captured by the primary case definition. This limitation means that pandemic-era coding patterns may still influence the comparison between MCOD-defined and underlying-cause-only mortality.

### 4.3 Age-stratified findings

The mortality burden was concentrated in older males. Men aged 65–85+ years had substantially higher AAMRs than those aged 45–64 years throughout the study period. This age gradient is expected, given the strong age dependence of prostate cancer incidence, metastatic progression, and mortality rates (1–3). The higher observed and projected burden in older men reflects both baseline disease risk and population aging, rather than any age-specific change in care or biology that this dataset can identify.

The projection results suggest that mortality counts in older age groups may rise, which has planning relevance for palliative care, home-based symptom management, radiotherapy access, bone-directed therapy, and community oncology support, although these service inferences are speculative extensions of the surveillance findings and not direct conclusions from the data. The place-of-death findings are relevant as a descriptive context: many deaths occurred at home or in hospice-related settings, consistent with broader US trends showing a shift in end-of-life care away from hospitals (33–35). Among patients with metastatic prostate cancer, the use of palliative care has increased over time, although disparities in its utilization persist (36).

### 4.4 Racial disparities

Non-Hispanic Black or African American men had the highest mortality burden in the MCOD-defined cohort. This finding is consistent with prior evidence showing persistent racial disparities in prostate cancer mortality and associations with social and neighborhood disadvantages (5, 6, 14, 15). However, the present ecological analysis cannot determine whether the observed disparity reflects differences in screening access, stage at diagnosis, treatment access, comorbidity, documentation practices, or a combination of these factors. Notably, although non-Hispanic Black or African American men had the highest observed mean AAMR, their overall 1999–2024 AAPC was not statistically significant, with the increase concentrated in specific joinpoint segments. Therefore, the observed data evidence for a disparity is strongest for absolute mean rates and recent segments, whereas the projected widening of the gap to 2040 rests on model-based extrapolation rather than directly observed full-period trends.

Evidence from the VA and equal-access healthcare settings suggests that some racial disparities in prostate cancer outcomes may narrow with improved access to timely diagnosis and treatment (37–40); however, these studies do not demonstrate that access barriers account for all of the observed differences in this MCOD analysis.

The projected widening of the non-Hispanic Black or African American to non-Hispanic White AAMRs ratio should be interpreted cautiously. This reflects the continuation of observed historical patterns under the selected forecasting models, not a deterministic prediction. These projected differences warrant continued monitoring and investigation of contributing factors in higher-burden populations; this dataset alone cannot identify what drives them or which interventions would alter them.

Halabi et al. offer useful clinical context by showing that metastatic site is associated with survival in men with advanced prostate cancer (41). However, because their analysis was based on castration-resistant prostate cancer trial populations and CDC WONDER lacks information on metastatic site combinations, disease stage, treatment exposure, and clinical course, these findings should be interpreted only as contextual background rather than as evidence explaining the population-level mortality trends observed in this study.

### 4.5 Geographic variation and urbanization

The analysis showed regional and urbanization-related differences in MCOD-defined mortality. The West had the highest mean AAMR over the study period and the steepest average annual increase, while the Midwest showed a particularly sharp acceleration after 2012. Non-metropolitan areas had higher mean AAMRs than metropolitan areas. These patterns are consistent with previous studies describing rural cancer care disparities, including differences in access to screening, specialty care, radiation oncology, imaging, and treatment services (42–44).

The interpretation of these results should be conservative. State-level and regional findings may reflect true geographic differences, but they may also be influenced by small numbers, population age structure, death certificate coding practices, and differences in local diagnostic documentation practices. Similarly, rural–urban rate differences are consistent with access-related explanations; however, this study cannot directly measure travel distance, physician density, insurance status, imaging availability, or treatment receipt, and no causal attribution is warranted from these data.

Therefore, the geographic results are best treated as surveillance signals identifying areas where advanced prostate cancer care capacity, palliative care access, and diagnostic pathways may warrant closer evaluation rather than as evidence linking any specific health-system factor to the observed mortality pattern.

### 4.6 Sensitivity analyses in context

The pre-pandemic sensitivity analysis indicated that the increasing MCOD-defined trend was already present before the major COVID-era disruptions. This supports the interpretation that the observed pattern was not solely an artifact of the pandemic. However, the pandemic period remains difficult to interpret because screening, biopsy, diagnosis, care delivery, and death certificate coding were all affected (23, 24, 32).

Chen et al. reported substantial reductions in cancer screening during the early pandemic, including prostate cancer screening (23). Englum et al. reported a reduction in the number of new cancer diagnoses and prostate biopsies within the VA system (24). These disruptions could have affected the diagnosis and documentation of metastatic disease. Therefore, the 2020–2024 portion of the trend should not be interpreted as a simple continuation of the pre-pandemic trajectory.

The underlying cause-only comparison indicates that conventional mortality surveillance and MCOD-defined surveillance answer different questions. Underlying cause-only data remain useful for tracking the standard mortality rate. MCOD data provide information on clinically important co-documented conditions, such as bone metastasis, but are more sensitive to documentation practices (4, 16, 25).

### 4.7 Strengths and limitations

This study used a national mortality database covering a long ICD-10-coded period, allowing trend assessment across age, race/ethnicity, region, and urbanization strata in the United States. The dual-field definition required prostate cancer as the underlying cause and bone metastasis among the multiple causes of death, creating a specific surveillance cohort rather than a broad any-mention prostate cancer sample (4, 16). Joinpoint regression and time-series forecasting were applied using standard methods for temporal and projection analyses (19–22).

This study had several limitations. First, death certificate data are vulnerable to misclassification of both the underlying and contributing causes of death (16, 25). Second, bone metastasis documentation may depend on the availability of imaging, clinician awareness, previous diagnosis, and institutional coding practices. Third, the cross-sectional surveillance design precludes individual-level inference and causal interpretation. Fourth, CDC WONDER does not provide patient-level treatment, comorbidity, socioeconomic, clinical stage, imaging, or recurrence data. Fifth, racial and ethnic classification on death certificates may be imperfect, particularly for some minority groups. Sixth, data suppression for small cell counts limited some of the subgroup analyses. Seventh, urbanization-stratified data were only available until 2020 using the relevant CDC WONDER classification (18).

Projection analyses also have their limitations. Forecasts assume that historical patterns contain useful information about future trends; however, prostate cancer is sensitive to screening policies, diagnostic technologies, treatment uptake, and coding changes. Li et al. showed that cancer projections can vary by method and that prostate cancer can be challenging to forecast because historical screening shifts introduce non-stationarity (22). Therefore, the 2040 estimates should be used as planning scenarios rather than precise future rates.

### 4.8 Implications

These findings suggest that MCOD-based measures may complement conventional prostate cancer mortality surveillance methods. Underlying-cause-only analyses describe the primary certified cause of death, whereas MCOD analyses provide additional information on clinically relevant co-documented conditions. For advanced prostate cancer, bone metastasis is especially relevant because it is closely associated with pain, skeletal events, radiotherapy needs, palliative care, and the place of death (11, 12, 33–36).

From a health system perspective, the higher mortality counts projected among older men are consistent with the growing demand for community-based palliative care, bone-directed therapies, pain management, and home-based support, although these remain planning inferences rather than direct findings (12, 36). The racial and rural–urban rate differences identified in this analysis warrant a focused investigation of contributing factors, including screening access, specialist referral patterns, staging, and treatment equity in populations identified as higher-burden in this dataset (14, 15, 37–40, 42–44). These surveillance signals require further investigation and should not be considered as evidence of specific causal pathways.

The global prostate cancer burden is expected to rise with population aging, and US findings should be interpreted within this broader demographic context (2, 3). Therefore, planning for advanced prostate cancer care should consider both conventional mortality indicators and MCOD-defined markers of metastatic complications.

## 5 Conclusion

Between 1999 and 2024, mortality records with prostate cancer as the underlying cause and bone metastasis co-documented among multiple causes of death increased in the United States. This pattern differed from the conventional underlying cause-of-death-only comparison, suggesting that MCOD surveillance may capture an important component of the advanced prostate cancer burden that is less visible in the standard mortality summaries. The observed increases were most pronounced among older men and were recorded at higher rates among non-Hispanic Black or African American men and those residing in non-metropolitan areas of the United States. Because these are death certificate-based measures, the observed trends reflect deaths with documented bone metastasis and may be affected by changes in coding and documentation practices; they should not be read as a direct measure of the true incidence of bone metastatic disease. Because this was a cross-sectional mortality surveillance study, the findings should not be interpreted as causal evidence linking screening policy, treatment uptake, imaging, or access barriers directly to mortality trends. Instead, they support continued MCOD-based monitoring, cautious interpretation of projection estimates, and targeted evaluation of advanced prostate cancer care needs in high-burden populations.

## Data Availability

The datasets presented in this study can be found in online repositories. The names of the repository/repositories and accession number(s) can be found in the article/**Supplementary Material**.

## References

[1] Islami F Arias G Lee D Wiese D Baeker Bispo J Yabroff KR . American Cancer Society’s report on the status of cancer disparities in the United States, 2025. CA Cancer J Clin. (2025) 76:e70045. doi: 10.3322/caac.70045 41400551 PMC12707308

[2] Shah R Battisti NM Brain E Gnangnon FH Kanesvaran R Mohile S . Updated cancer burden in oldest old: a population-based study using 2022 Globocan estimates. Cancer Epidemiol. (2025) 95:102716. doi: 10.1016/j.canep.2024.102716 39603975

[3] James ND Tannock I N'Dow J Feng F Gillessen S Ali SA . The Lancet Commission on prostate cancer: planning for the surge in cases. Lancet. (2024) 403:1683–722. doi: 10.1016/S0140-6736(24)00651-2 38583453 PMC7617369

[4] Centers for Disease Control and Prevention, National Center for Health Statistics . Multiple Cause of Death 1999–2024 on CDC WONDER Online Database. Atlanta: US Department of Health and Human Services (2024). Available online at: https://wonder.cdc.gov/mcd-icd10-expanded.html (Accessed April 1, 2026).

[5] Chai Z Yan S Li H Dong X Li S Fan Y . Temporal trends and regional disparities in prostate cancer mortality in the United States, 1999–2023: an analysis of the CDC WONDER database. BMC Public Health. (2025) 25:4263. doi: 10.1186/s12889-025-25694-6 41413487 PMC12713269

[6] Ghazwani Y Alghafees M Suheb MK Shafqat A Sabbah BN Arabi TZ . Trends in genitourinary cancer mortality in the United States: analysis of the CDC-WONDER database 1999–2020. Front Public Health. (2024) 12:1354663. doi: 10.3389/fpubh.2024.1354663 38966707 PMC11223728

[7] Drazer MW Huo D Eggener SE . National prostate cancer screening rates after the 2012 US Preventive Services Task Force recommendation discouraging prostate-specific antigen-based screening. J Clin Oncol. (2015) 33:2416–23. doi: 10.1200/JCO.2015.61.6532 26056181

[8] Burgess L Aldrighetti CM Ghosh A Niemierko A Chino F Huynh MJ . Association of the USPSTF Grade D recommendation against prostate-specific antigen screening with prostate cancer–specific mortality. JAMA Netw Open. (2022) 5:e2211869. doi: 10.1001/jamanetworkopen.2022.11869 35576008 PMC9112070

[9] Desai MM Cacciamani GE Gill K Zhang J Liu L Abreu A . Trends in incidence of metastatic prostate cancer in the US. JAMA Netw Open. (2022) 5:e222246. doi: 10.1001/jamanetworkopen.2022.2246 35285916 PMC9907338

[10] Long N Woodlock D D’Agostino R Nguyen G Gangai N Sevilimedu V . Incidence and prevalence of bone metastases in different solid tumors determined by natural language processing of CT reports. Cancers (Basel). (2025) 17:218. doi: 10.3390/cancers17020218 39858000 PMC11763382

[11] Guo X Li S . Bone metastases of prostate cancer: molecular mechanisms, targeted diagnosis and targeted therapy (Review). Oncol Rep. (2025) 53:46. doi: 10.3892/or.2025.8879 39981932 PMC11865881

[12] McDougall JA Bansal A Goulart BHL McCune JS Karnopp A Fedorenko C . The clinical and economic impacts of skeletal-related events among Medicare enrollees with prostate cancer metastatic to bone. Oncologist. (2016) 21:320–6. doi: 10.1634/theoncologist.2015-0327 26865591 PMC4786354

[13] National Cancer Institute . SEER Cancer Stat Facts: Prostate Cancer. Bethesda: NIH (2025). Available online at: https://seer.cancer.gov/statfacts/html/common.html (Accessed April 1, 2026).

[14] Press DJ Shariff-Marco S Lichtensztajn DY Lauderdale D Murphy AB Inamdar PP . Contributions of social factors to disparities in prostate cancer risk profiles among Black men and Non-Hispanic White men with prostate cancer in California. Cancer Epidemiol Biomarkers Prev. (2022) 31:404–12. doi: 10.1158/1055-9965.EPI-21-0697 34853020 PMC8825684

[15] Iyer HS Gomez SL Chen JT Trinh QD Rebbeck TR . Trends in mortality among Black and White men with prostate cancer in Massachusetts and Pennsylvania: race and neighborhood socioeconomic position. Cancer. (2021) 127:2525–34. doi: 10.1002/cncr.33506 33798264 PMC8249310

[16] Minhas AMK Sperling LS Al-Kindi S Abramov D . Underlying and contributing causes of mortality from CDC WONDER — insights for researchers. Am Heart J Plus. (2025) 50:100499. doi: 10.1016/j.ahjo.2025.100499 39895921 PMC11782113

[17] von Elm E Altman DG Egger M Pocock SJ Gøtzsche PC Vandenbroucke JP . The Strengthening the Reporting of Observational Studies in Epidemiology (STROBE) statement: guidelines for reporting observational studies. Lancet. (2007) 370:1453–7. doi: 10.1016/S0140-6736(07)61602-X 18064739

[18] Ingram DD Franco SJ . 2013 NCHS urban-rural classification scheme for counties. Vital Health Stat 2. (2014), 1–73 24776070

[19] National Cancer Institute . Joinpoint Trend Analysis Software, Version 5.2.0. Bethesda: National Cancer Institute (2023). Available online at: https://surveillance.cancer.gov/joinpoint/ (Accessed April 1, 2026).

[20] Miller KD Siegel RL Liu B Zhu L Zou J Jemal A . Updated methodology for projecting US- and state-level cancer counts, part II: evaluation of incidence and mortality projection methods. Cancer Epidemiol Biomarkers Prev. (2021) 30:1993–2000. doi: 10.1158/1055-9965.EPI-20-1780 34404684

[21] Earnest A Evans SM Sampurno F Millar J . Forecasting annual incidence and mortality rate for prostate cancer in Australia until 2022 using ARIMA models. BMJ Open. (2019) 9:e031331. doi: 10.1136/bmjopen-2019-031331 31431447 PMC6707661

[22] Li J Chan NB Xue J Tsoi KKF . Time series models show comparable projection performance with joinpoint regression: a comparison using historical cancer data from WHO. Front Public Health. (2022) 10:1003162. doi: 10.3389/fpubh.2022.1003162 36311591 PMC9614249

[23] Chen RC Haynes K Du S Barron J Katz AJ . Association of cancer screening deficit in the United States with the COVID-19 pandemic. JAMA Oncol. (2021) 7:878–84. doi: 10.1001/jamaoncol.2021.0884 33914015 PMC8085759

[24] Englum BR Prasad NK Lake RE Mayorga-Carlin M Turner DJ Siddiqui T . Impact of the COVID-19 pandemic on diagnosis of new cancers: a national multicenter study of the Veterans Affairs Healthcare System. Cancer. (2022) 128:1048–56. doi: 10.1002/cncr.34011 34866184 PMC8837676

[25] Turner EL Metcalfe C Donovan JL Noble S Sterne JAC Lane JA . Contemporary accuracy of death certificates for coding prostate cancer as a cause of death. Br J Cancer. (2016) 115:90–4. doi: 10.1038/bjc.2016.162 27253172 PMC4931376

[26] Fizazi K Tran N Fein L Matsubara N Rodriguez-Antolin A Alekseev BY . Abiraterone acetate plus prednisone in newly diagnosed high-risk metastatic castration-sensitive prostate cancer: final overall survival analysis of LATITUDE. Lancet Oncol. (2019) 20:686–700. doi: 10.1016/S1470-2045(19)30082-8 30987939

[27] Armstrong AJ Azad AA Iguchi T Szmulewitz RZ Petrylak DP Holzbeierlein J . Improved survival with enzalutamide in patients with metastatic hormone-sensitive prostate cancer. J Clin Oncol. (2022) 40:1616–22. doi: 10.1200/JCO.22.00193 35420921 PMC9113211

[28] Chi KN Agarwal N Bjartell A Chung BH Pereira de Santana Gomes AJ Given R . Apalutamide for metastatic, castration-sensitive prostate cancer. N Engl J Med. (2019) 381:13–24. doi: 10.1056/nejmoa1903307 31150574

[29] Smith MR Hussain M Saad F Fizazi K Sternberg CN Crawford ED . Darolutamide and survival in metastatic, hormone-sensitive prostate cancer. N Engl J Med. (2022) 386:1132–42. doi: 10.1056/nejmoa2119115 35179323 PMC9844551

[30] Hofman MS Lawrentschuk N Francis RJ Tang C Vela I Thomas P . Prostate-specific membrane antigen PET-CT in patients with high-risk prostate cancer before curative-intent surgery or radiotherapy (proPSMA): a prospective, randomised, multicentre study. Lancet. (2020) 395:1208–16. doi: 10.1016/s0140-6736(20)30314-7 32209449

[31] Chow KM So WZ Lee HJ Lee A Yap DW Takwoingi Y . Head-to-head comparison of the diagnostic accuracy of prostate-specific membrane antigen positron emission tomography and conventional imaging modalities for initial staging of intermediate-to high-risk prostate cancer: a systematic review and meta-analysis. Eur Urol. (2023) 84:36–48. doi: 10.1016/j.eururo.2023.03.001 37032189

[32] Hansen CL Viboud C Simonsen L . Disentangling the relationship between cancer mortality and COVID-19 in the US. eLife. (2024) 13:RP93758. doi: 10.7554/eLife.93758 39190600 PMC11349294

[33] Cross SH Warraich HJ . Changes in the place of death in the United States. N Engl J Med. (2019) 381:2369–70. doi: 10.1056/NEJMc1911892 31826345

[34] Cross SH Kaufman BG Quest TE Warraich HJ . National trends in hospice facility deaths in the United States, 2003–2017. J Pain Symptom Manage. (2021) 61:350–7. doi: 10.1016/j.jpainsymman.2020.08.026 32858165

[35] Aldridge MD Ornstein KA McKendrick K Moreno J Reckrey JM Li L . Trends in residential setting and hospice use at the end of life for Medicare decedents. Health Aff (Millwood). (2020) 39:1060–4. doi: 10.1377/hlthaff.2019.01549 32479223 PMC8045974

[36] Olafimihan A Jackson I Nwachukwu C Ozogbo S Oh Y George L . Trends, sociodemographic and hospital-level factors associated with palliative care utilization among metastatic prostate cancer patients. Am J Hosp Palliat Care. (2025) 42:404–12. doi: 10.1177/10499091241256627 38780478

[37] Yamoah K Lee KM Awasthi S Alba PR Perez C Anglin-Foote TR . Racial and ethnic disparities in prostate cancer outcomes in the Veterans Affairs Health Care System. JAMA Netw Open. (2022) 5:e2144027. doi: 10.1001/jamanetworkopen.2021.44027 35040965 PMC8767437

[38] Lillard JW Moses KA Mahal BA George DJ . Racial disparities in Black men with prostate cancer: a literature review. Cancer. (2022) 128:3787–95. doi: 10.1002/cncr.34433 36066378 PMC9826514

[39] Dess RT Hartman HE Mahal BA Soni PD Jackson WC Cooperberg MR . Association of Black race with prostate cancer–specific and other-cause mortality. JAMA Oncol. (2019) 5:975–83. doi: 10.1001/jamaoncol.2019.0826 31120534 PMC6547116

[40] Riviere P Luterstein E Kumar A Vitzthum LK Deka R Sarkar RR . Survival of African American and non-Hispanic white men with prostate cancer in an equal-access health care system. Cancer. (2020) 126:1683–90. doi: 10.1002/cncr.32666 31984482

[41] Halabi S Kelly WK Ma H Zhou H Solomon NC Fizazi K . Meta-analysis evaluating the impact of site of metastasis on overall survival in men with castration-resistant prostate cancer. J Clin Oncol. (2016) 34:1652–9. doi: 10.1200/JCO.2015.65.7270 26951312 PMC4872320

[42] Bhatia S Landier W Paskett ED Peters KB Merrill JK Phillips J . Rural-urban disparities in cancer outcomes: opportunities for future research. J Natl Cancer Inst. (2022) 114:940–52. doi: 10.1093/jnci/djac030 35148389 PMC9275775

[43] Maganty A Sabik LM Sun Z Eom KY Li J Davies BJ . Undertreatment of prostate cancer in rural locations. J Urol. (2020) 203:108–14. doi: 10.1097/JU.0000000000000500 31430233 PMC7098431

[44] Blake KD Moss JL Gaysynsky A Srinivasan S Croyle RT . Making the case for investment in rural cancer control. Cancer Epidemiol Biomarkers Prev. (2017) 26:992–7. doi: 10.1158/1055-9965.EPI-17-0092 28600296 PMC5500425

