# Supplementary material for: National trends in bone-metastatic prostate cancer mortality in the United States, 1999–2024: a multiple-cause-of-death analysis with projections to 2040
Source: Front Oncol. 2026 Jul 20;16:1882666. doi: 10.3389/fonc.2026.1882666 (PMC13429431; doi:10.3389/fonc.2026.1882666)
Supplement: Supplementary file 1 [file Table1.docx]

**National Trends in Bone-Metastatic Prostate Cancer Mortality in the United States, 1999-2024: A Multiple-Cause-of-Death Analysis With Projections to 2040**

**Supplementary Tables**

**Supplementary Table 1. Overall annual mortality trends and age-adjusted mortality rates, 1999-2024.**

| **Cohort** | **Year** | **Deaths** | **Population** | **Age-adjusted mortality rate per 100,000 (95% CI)** |
| --- | --- | --- | --- | --- |
| Overall | 1999 | 1949 | 180408769 | 1.10 (1.05–1.15) |
| Overall | 2000 | 1867 | 181984640 | 1.05 (1.00–1.10) |
| Overall | 2001 | 1781 | 184305128 | 0.99 (0.94–1.03) |
| Overall | 2002 | 1697 | 186208028 | 0.92 (0.88–0.96) |
| Overall | 2003 | 1553 | 188090429 | 0.84 (0.80–0.88) |
| Overall | 2004 | 1543 | 190205384 | 0.82 (0.78–0.86) |
| Overall | 2005 | 1567 | 192551384 | 0.82 (0.78–0.86) |
| Overall | 2006 | 1451 | 195019359 | 0.74 (0.70–0.78) |
| Overall | 2007 | 1573 | 197403777 | 0.80 (0.76–0.84) |
| Overall | 2008 | 1409 | 199795090 | 0.70 (0.67–0.74) |
| Overall | 2009 | 1604 | 202107016 | 0.78 (0.74–0.82) |
| Overall | 2010 | 1771 | 203891983 | 0.85 (0.81–0.89) |
| Overall | 2011 | 1876 | 206592936 | 0.87 (0.83–0.91) |
| Overall | 2012 | 1887 | 208826037 | 0.84 (0.80–0.88) |
| Overall | 2013 | 2127 | 211085314 | 0.93 (0.89–0.97) |
| Overall | 2014 | 2337 | 213809280 | 1.00 (0.96–1.04) |
| Overall | 2015 | 2676 | 216553817 | 1.12 (1.08–1.16) |
| Overall | 2016 | 3351 | 218641417 | 1.34 (1.30–1.39) |
| Overall | 2017 | 3765 | 221447331 | 1.48 (1.43–1.53) |
| Overall | 2018 | 4278 | 223311190 | 1.63 (1.58–1.68) |
| Overall | 2019 | 4531 | 224981167 | 1.68 (1.63–1.73) |
| Overall | 2020 | 4864 | 226635013 | 1.78 (1.73–1.83) |
| Overall | 2021 | 5049 | 224030595 | 1.93 (1.87–1.98) |
| Overall | 2022 | 5289 | 225125435 | 1.89 (1.84–1.95) |
| Overall | 2023 | 5551 | 226995452 | 1.99 (1.94–2.04) |
| Overall | 2024 | 6083 | 230831890 | 2.12 (2.06–2.17) |

*Abbreviations: AAMR, age-adjusted mortality rate; CI, confidence interval. Note: AAMRs are reported per 100,000 population.*

**Supplementary Table 2. Projected age-adjusted mortality rates for the overall male-only cohort, 2025-2040.**

| **Cohort** | **Year** | **ETS Age-adjusted mortality rate per 100,000 (95% CI)** |
| --- | --- | --- |
| Overall | 2025 | 2.29 (2.06–2.52) |
| Overall | 2026 | 2.38 (2.05–2.71) |
| Overall | 2027 | 2.47 (2.03–2.91) |
| Overall | 2028 | 2.56 (1.99–3.13) |
| Overall | 2029 | 2.65 (1.94–3.36) |
| Overall | 2030 | 2.74 (1.89–3.60) |
| Overall | 2031 | 2.83 (1.82–3.85) |
| Overall | 2032 | 2.92 (1.75–4.10) |
| Overall | 2033 | 3.02 (1.66–4.37) |
| Overall | 2034 | 3.11 (1.57–4.64) |
| Overall | 2035 | 3.20 (1.47–4.92) |
| Overall | 2036 | 3.29 (1.37–5.21) |
| Overall | 2037 | 3.38 (1.26–5.51) |
| Overall | 2038 | 3.47 (1.14–5.81) |
| Overall | 2039 | 3.56 (1.01–6.12) |
| Overall | 2040 | 3.65 (0.88–6.43) |

*Abbreviations: AAMR, age-adjusted mortality rate; CI, confidence interval; ETS, error, trend, seasonal model. Note: Forecasted AAMRs are model-based projections and are reported per 100,000 population.*

**Supplementary Table 3. Annual mortality trends and age-adjusted mortality rates by age group, 1999-2024.**

| **Age Group** | **Year** | **Deaths** | **Population** | **Age-adjusted mortality rate per 100,000 (95% CI)** |
| --- | --- | --- | --- | --- |
| 45-64 | 1999 | 176 | 60355845 | 0.30 (0.25–0.34) |
| 45-64 | 2000 | 154 | 61952636 | 0.26 (0.22–0.30) |
| 45-64 | 2001 | 177 | 64491563 | 0.30 (0.25–0.34) |
| 45-64 | 2002 | 153 | 66695526 | 0.22 (0.18–0.25) |
| 45-64 | 2003 | 151 | 68828899 | 0.26 (0.21–0.30) |
| 45-64 | 2004 | 172 | 70935234 | 0.26 (0.22–0.30) |
| 45-64 | 2005 | 179 | 73137401 | 0.26 (0.22–0.30) |
| 45-64 | 2006 | 161 | 75216272 | 0.22 (0.18–0.25) |
| 45-64 | 2007 | 182 | 77068373 | 0.26 (0.22–0.30) |
| 45-64 | 2008 | 161 | 78617510 | 0.22 (0.18–0.25) |
| 45-64 | 2009 | 208 | 80272688 | 0.26 (0.22–0.29) |
| 45-64 | 2010 | 238 | 81489445 | 0.30 (0.26–0.34) |
| 45-64 | 2011 | 247 | 82780343 | 0.30 (0.26–0.33) |
| 45-64 | 2012 | 250 | 82854940 | 0.26 (0.22–0.29) |
| 45-64 | 2013 | 269 | 83083963 | 0.30 (0.26–0.33) |
| 45-64 | 2014 | 269 | 83536432 | 0.30 (0.26–0.33) |
| 45-64 | 2015 | 356 | 84065980 | 0.38 (0.34–0.41) |
| 45-64 | 2016 | 391 | 84249823 | 0.38 (0.34–0.41) |
| 45-64 | 2017 | 440 | 84370610 | 0.41 (0.38–0.45) |
| 45-64 | 2018 | 534 | 83904335 | 0.49 (0.45–0.53) |
| 45-64 | 2019 | 521 | 83323439 | 0.49 (0.45–0.54) |
| 45-64 | 2020 | 553 | 82769810 | 0.53 (0.49–0.58) |
| 45-64 | 2021 | 564 | 82231945 | 0.59 (0.55–0.64) |
| 45-64 | 2022 | 527 | 81224314 | 0.49 (0.45–0.54) |
| 45-64 | 2023 | 579 | 81027312 | 0.59 (0.54–0.64) |
| 45-64 | 2024 | 565 | 81072359 | 0.59 (0.55–0.64) |
| 65-85+ | 1999 | 1771 | 34797841 | 5.09 (4.86–5.33) |
| 65-85+ | 2000 | 1713 | 34991753 | 4.92 (4.68–5.15) |
| 65-85+ | 2001 | 1601 | 35290291 | 4.54 (4.31–4.76) |
| 65-85+ | 2002 | 1540 | 35522207 | 4.33 (4.11–4.55) |
| 65-85+ | 2003 | 1401 | 35863529 | 3.86 (3.66–4.06) |
| 65-85+ | 2004 | 1370 | 36203319 | 3.76 (3.56–3.95) |
| 65-85+ | 2005 | 1383 | 36649798 | 3.74 (3.55–3.94) |
| 65-85+ | 2006 | 1289 | 37164107 | 3.42 (3.23–3.61) |
| 65-85+ | 2007 | 1391 | 37825711 | 3.66 (3.47–3.85) |
| 65-85+ | 2008 | 1248 | 38777621 | 3.21 (3.03–3.39) |
| 65-85+ | 2009 | 1395 | 39623175 | 3.55 (3.36–3.74) |
| 65-85+ | 2010 | 1532 | 40267984 | 3.81 (3.61–4.00) |
| 65-85+ | 2011 | 1627 | 41394141 | 3.92 (3.73–4.11) |
| 65-85+ | 2012 | 1634 | 43145356 | 3.84 (3.66–4.03) |
| 65-85+ | 2013 | 1856 | 44704074 | 4.21 (4.02–4.41) |
| 65-85+ | 2014 | 2067 | 46243211 | 4.60 (4.40–4.80) |
| 65-85+ | 2015 | 2317 | 47760852 | 5.08 (4.87–5.29) |
| 65-85+ | 2016 | 2957 | 49244195 | 6.21 (5.98–6.43) |
| 65-85+ | 2017 | 3322 | 50858679 | 6.86 (6.62–7.09) |
| 65-85+ | 2018 | 3741 | 52431193 | 7.48 (7.24–7.73) |
| 65-85+ | 2019 | 4006 | 54058263 | 7.74 (7.50–7.99) |
| 65-85+ | 2020 | 4307 | 55659365 | 8.15 (7.91–8.40) |
| 65-85+ | 2021 | 4483 | 55286556 | 8.82 (8.56–9.08) |
| 65-85+ | 2022 | 4758 | 57186991 | 8.83 (8.58–9.09) |
| 65-85+ | 2023 | 4969 | 58612299 | 9.13 (8.88–9.39) |
| 65-85+ | 2024 | 5513 | 60500815 | 9.79 (9.53–10.05) |

*Abbreviations: AAMR, age-adjusted mortality rate; CI, confidence interval. Note: Age strata shown are 45-64 years and 65-85+ years; deaths in younger age groups are not displayed in this table, so age-stratified deaths do not sum to the overall total.*

**Supplementary Table 4. Projected age-adjusted mortality rates by age group, 2025-2040.**

| **Age** | **Year** | **Age-adjusted mortality rate per 100,000 (95% CI)** | **Forecast model** |
| --- | --- | --- | --- |
| 45-64 years | 2025 | 0.62 (0.52–0.71) | ARIMA |
| 45-64 years | 2026 | 0.63 (0.52–0.75) | ARIMA |
| 45-64 years | 2027 | 0.64 (0.52–0.77) | ARIMA |
| 45-64 years | 2028 | 0.66 (0.52–0.80) | ARIMA |
| 45-64 years | 2029 | 0.67 (0.52–0.82) | ARIMA |
| 45-64 years | 2030 | 0.68 (0.52–0.84) | ARIMA |
| 45-64 years | 2031 | 0.69 (0.52–0.87) | ARIMA |
| 45-64 years | 2032 | 0.71 (0.52–0.89) | ARIMA |
| 45-64 years | 2033 | 0.72 (0.53–0.91) | ARIMA |
| 45-64 years | 2034 | 0.73 (0.53–0.93) | ARIMA |
| 45-64 years | 2035 | 0.74 (0.54–0.95) | ARIMA |
| 45-64 years | 2036 | 0.76 (0.54–0.97) | ARIMA |
| 45-64 years | 2037 | 0.77 (0.55–0.99) | ARIMA |
| 45-64 years | 2038 | 0.78 (0.55–1.01) | ARIMA |
| 45-64 years | 2039 | 0.79 (0.56–1.03) | ARIMA |
| 45-64 years | 2040 | 0.81 (0.56–1.05) | ARIMA |
| 65-85+ years | 2025 | 10.60 (9.56–11.63) | ETS |
| 65-85+ years | 2026 | 11.03 (9.52–12.55) | ETS |
| 65-85+ years | 2027 | 11.47 (9.42–13.52) | ETS |
| 65-85+ years | 2028 | 11.91 (9.26–14.55) | ETS |
| 65-85+ years | 2029 | 12.34 (9.05–15.63) | ETS |
| 65-85+ years | 2030 | 12.78 (8.80–16.76) | ETS |
| 65-85+ years | 2031 | 13.21 (8.50–17.93) | ETS |
| 65-85+ years | 2032 | 13.65 (8.16–19.14) | ETS |
| 65-85+ years | 2033 | 14.09 (7.78–20.39) | ETS |
| 65-85+ years | 2034 | 14.52 (7.37–21.68) | ETS |
| 65-85+ years | 2035 | 14.96 (6.92–23.00) | ETS |
| 65-85+ years | 2036 | 15.39 (6.44–24.35) | ETS |
| 65-85+ years | 2037 | 15.83 (5.92–25.74) | ETS |
| 65-85+ years | 2038 | 16.27 (5.37–27.16) | ETS |
| 65-85+ years | 2039 | 16.70 (4.79–28.61) | ETS |
| 65-85+ years | 2040 | 17.14 (4.19–30.09) | ETS |

*Abbreviations: AAMR, age-adjusted mortality rate; ARIMA, autoregressive integrated moving average; CI, confidence interval; ETS, error, trend, seasonal model. Note: Forecasted AAMRs are model-based projections and are reported per 100,000 population.*

**Supplementary Table 5. Annual mortality trends and age-adjusted mortality rates by race/ethnicity, 1999-2024.**

| **Race** | **Year** | **Deaths** | **Population** | **Age-adjusted mortality rate per 100,000 (95% CI)** |
| --- | --- | --- | --- | --- |
| Hispanic or Latino | 1999 | 100 | 17503631 | 1.23 (0.98–1.48) |
| Hispanic or Latino | 2000 | 87 | 18219679 | 1.06 (0.84–1.31) |
| Hispanic or Latino | 2001 | 85 | 19290018 | 0.96 (0.76–1.19) |
| Hispanic or Latino | 2002 | 80 | 20159630 | 0.86 (0.67–1.07) |
| Hispanic or Latino | 2003 | 71 | 21011656 | 0.74 (0.58–0.94) |
| Hispanic or Latino | 2004 | 84 | 21877214 | 0.85 (0.67–1.06) |
| Hispanic or Latino | 2005 | 68 | 22804023 | 0.63 (0.48–0.80) |
| Hispanic or Latino | 2006 | 84 | 23743864 | 0.75 (0.60–0.94) |
| Hispanic or Latino | 2007 | 100 | 24673919 | 0.81 (0.65–0.97) |
| Hispanic or Latino | 2008 | 81 | 25602850 | 0.60 (0.47–0.75) |
| Hispanic or Latino | 2009 | 97 | 26504021 | 0.74 (0.59–0.90) |
| Hispanic or Latino | 2010 | 101 | 27192663 | 0.70 (0.56–0.84) |
| Hispanic or Latino | 2011 | 139 | 28255675 | 0.91 (0.76–1.07) |
| Hispanic or Latino | 2012 | 136 | 28988437 | 0.84 (0.69–0.98) |
| Hispanic or Latino | 2013 | 155 | 29784174 | 0.91 (0.76–1.06) |
| Hispanic or Latino | 2014 | 160 | 30809714 | 0.86 (0.72–1.00) |
| Hispanic or Latino | 2015 | 242 | 31761872 | 1.24 (1.08–1.40) |
| Hispanic or Latino | 2016 | 248 | 32438262 | 1.24 (1.08–1.40) |
| Hispanic or Latino | 2017 | 276 | 33594503 | 1.33 (1.17–1.49) |
| Hispanic or Latino | 2018 | 310 | 34350362 | 1.39 (1.23–1.55) |
| Hispanic or Latino | 2019 | 359 | 35025850 | 1.56 (1.40–1.73) |
| Hispanic or Latino | 2020 | 347 | 35758193 | 1.41 (1.25–1.56) |
| Hispanic or Latino | 2021 | 378 | 35806297 | 1.55 (1.40–1.72) |
| Hispanic or Latino | 2022 | 424 | 36463229 | 1.66 (1.50–1.83) |
| Hispanic or Latino | 2023 | 418 | 37642932 | 1.55 (1.40–1.71) |
| Hispanic or Latino | 2024 | 458 | 39459545 | 1.62 (1.47–1.78) |
| Non-Hispanic Black or African American | 1999 | 352 | 19808077 | 2.47 (2.21–2.73) |
| Non-Hispanic Black or African American | 2000 | 288 | 20058273 | 2.00 (1.77–2.23) |
| Non-Hispanic Black or African American | 2001 | 283 | 20404945 | 1.92 (1.69–2.15) |
| Non-Hispanic Black or African American | 2002 | 260 | 20695836 | 1.74 (1.53–1.96) |
| Non-Hispanic Black or African American | 2003 | 240 | 20982115 | 1.58 (1.38–1.78) |
| Non-Hispanic Black or African American | 2004 | 228 | 21327519 | 1.48 (1.29–1.68) |
| Non-Hispanic Black or African American | 2005 | 237 | 21703691 | 1.50 (1.31–1.70) |
| Non-Hispanic Black or African American | 2006 | 205 | 22092072 | 1.27 (1.09–1.44) |
| Non-Hispanic Black or African American | 2007 | 243 | 22474128 | 1.43 (1.25–1.61) |
| Non-Hispanic Black or African American | 2008 | 202 | 22857307 | 1.18 (1.01–1.35) |
| Non-Hispanic Black or African American | 2009 | 253 | 23236715 | 1.44 (1.26–1.62) |
| Non-Hispanic Black or African American | 2010 | 267 | 23537629 | 1.48 (1.30–1.67) |
| Non-Hispanic Black or African American | 2011 | 294 | 23958352 | 1.54 (1.36–1.72) |
| Non-Hispanic Black or African American | 2012 | 267 | 24345607 | 1.37 (1.20–1.54) |
| Non-Hispanic Black or African American | 2013 | 282 | 24743381 | 1.34 (1.18–1.51) |
| Non-Hispanic Black or African American | 2014 | 296 | 25244585 | 1.39 (1.23–1.55) |
| Non-Hispanic Black or African American | 2015 | 368 | 25752287 | 1.70 (1.53–1.88) |
| Non-Hispanic Black or African American | 2016 | 446 | 26212105 | 1.91 (1.72–2.09) |
| Non-Hispanic Black or African American | 2017 | 491 | 26762620 | 2.02 (1.84–2.21) |
| Non-Hispanic Black or African American | 2018 | 589 | 27178229 | 2.42 (2.22–2.62) |
| Non-Hispanic Black or African American | 2019 | 664 | 27592123 | 2.59 (2.39–2.80) |
| Non-Hispanic Black or African American | 2020 | 641 | 27969928 | 2.38 (2.19–2.57) |
| Non-Hispanic Black or African American | 2021 | 639 | 27538737 | 2.41 (2.22–2.61) |
| Non-Hispanic Black or African American | 2022 | 708 | 27685911 | 2.69 (2.49–2.90) |
| Non-Hispanic Black or African American | 2023 | 789 | 28042483 | 2.92 (2.71–3.14) |
| Non-Hispanic Black or African American | 2024 | 885 | 28588970 | 3.09 (2.89–3.31) |
| Non-Hispanic White | 1999 | 1478 | 134935890 | 1.00 (0.95–1.05) |
| Non-Hispanic White | 2000 | 1474 | 135202971 | 0.98 (0.93–1.03) |
| Non-Hispanic White | 2001 | 1394 | 135651679 | 0.91 (0.87–0.96) |
| Non-Hispanic White | 2002 | 1338 | 136026843 | 0.87 (0.82–0.91) |
| Non-Hispanic White | 2003 | 1220 | 136415868 | 0.77 (0.73–0.81) |
| Non-Hispanic White | 2004 | 1206 | 136956601 | 0.77 (0.73–0.82) |
| Non-Hispanic White | 2005 | 1244 | 137614452 | 0.79 (0.75–0.84) |
| Non-Hispanic White | 2006 | 1130 | 138363022 | 0.69 (0.65–0.74) |
| Non-Hispanic White | 2007 | 1198 | 139060265 | 0.75 (0.71–0.79) |
| Non-Hispanic White | 2008 | 1107 | 139772453 | 0.67 (0.63–0.71) |
| Non-Hispanic White | 2009 | 1227 | 140451863 | 0.74 (0.69–0.78) |
| Non-Hispanic White | 2010 | 1361 | 140987505 | 0.77 (0.73–0.81) |
| Non-Hispanic White | 2011 | 1406 | 141789725 | 0.81 (0.77–0.86) |
| Non-Hispanic White | 2012 | 1441 | 142424668 | 0.80 (0.76–0.84) |
| Non-Hispanic White | 2013 | 1647 | 143045234 | 0.91 (0.86–0.95) |
| Non-Hispanic White | 2014 | 1822 | 143642265 | 0.98 (0.94–1.03) |
| Non-Hispanic White | 2015 | 1984 | 144347874 | 1.05 (1.01–1.10) |
| Non-Hispanic White | 2016 | 2566 | 144979180 | 1.33 (1.28–1.39) |
| Non-Hispanic White | 2017 | 2906 | 145456222 | 1.49 (1.44–1.55) |
| Non-Hispanic White | 2018 | 3250 | 145801376 | 1.64 (1.58–1.69) |
| Non-Hispanic White | 2019 | 3374 | 146079678 | 1.64 (1.58–1.70) |
| Non-Hispanic White | 2020 | 3716 | 146337256 | 1.79 (1.73–1.85) |
| Non-Hispanic White | 2021 | 3882 | 144655323 | 1.97 (1.90–2.03) |
| Non-Hispanic White | 2022 | 4011 | 144499701 | 1.90 (1.84–1.96) |
| Non-Hispanic White | 2023 | 4168 | 144415042 | 2.00 (1.93–2.06) |
| Non-Hispanic White | 2024 | 4575 | 144849472 | 2.12 (2.06–2.18) |

*Abbreviations: AAMR, age-adjusted mortality rate; CI, confidence interval. Note: Race/ethnicity categories are reported as extracted from CDC WONDER. The categories shown are not exhaustive; therefore, race/ethnicity-specific deaths do not sum to the overall total.*

**Supplementary Table 6. Projected age-adjusted mortality rates by race/ethnicity, 2025-2040.**

| **Race** | **Year** | **Age-adjusted mortality rate per 100,000 (95% CI)** | **Forecast model** |
| --- | --- | --- | --- |
| Hispanic or Latino | 2025 | 1.61 (1.32–1.91) | ARIMA |
| Hispanic or Latino | 2026 | 1.62 (1.22–2.01) | ARIMA |
| Hispanic or Latino | 2027 | 1.62 (1.09–2.16) | ARIMA |
| Hispanic or Latino | 2028 | 1.63 (0.93–2.33) | ARIMA |
| Hispanic or Latino | 2029 | 1.64 (0.75–2.53) | ARIMA |
| Hispanic or Latino | 2030 | 1.64 (0.55–2.74) | ARIMA |
| Hispanic or Latino | 2031 | 1.65 (0.32–2.98) | ARIMA |
| Hispanic or Latino | 2032 | 1.66 (0.09–3.23) | ARIMA |
| Hispanic or Latino | 2033 | 1.66 (0.00–3.49) | ARIMA |
| Hispanic or Latino | 2034 | 1.67 (0.00–3.77) | ARIMA |
| Hispanic or Latino | 2035 | 1.68 (0.00–4.06) | ARIMA |
| Hispanic or Latino | 2036 | 1.69 (0.00–4.36) | ARIMA |
| Hispanic or Latino | 2037 | 1.69 (0.00–4.68) | ARIMA |
| Hispanic or Latino | 2038 | 1.70 (0.00–5.00) | ARIMA |
| Hispanic or Latino | 2039 | 1.71 (0.00–5.34) | ARIMA |
| Hispanic or Latino | 2040 | 1.71 (0.00–5.69) | ARIMA |
| Non-Hispanic Black or African American | 2025 | 3.41 (2.77–4.04) | ARIMA |
| Non-Hispanic Black or African American | 2026 | 3.56 (2.69–4.44) | ARIMA |
| Non-Hispanic Black or African American | 2027 | 3.72 (2.59–4.85) | ARIMA |
| Non-Hispanic Black or African American | 2028 | 3.88 (2.48–5.28) | ARIMA |
| Non-Hispanic Black or African American | 2029 | 4.04 (2.36–5.71) | ARIMA |
| Non-Hispanic Black or African American | 2030 | 4.19 (2.22–6.17) | ARIMA |
| Non-Hispanic Black or African American | 2031 | 4.35 (2.07–6.63) | ARIMA |
| Non-Hispanic Black or African American | 2032 | 4.51 (1.91–7.11) | ARIMA |
| Non-Hispanic Black or African American | 2033 | 4.67 (1.73–7.61) | ARIMA |
| Non-Hispanic Black or African American | 2034 | 4.83 (1.54–8.11) | ARIMA |
| Non-Hispanic Black or African American | 2035 | 4.98 (1.34–8.63) | ARIMA |
| Non-Hispanic Black or African American | 2036 | 5.14 (1.13–9.16) | ARIMA |
| Non-Hispanic Black or African American | 2037 | 5.30 (0.90–9.70) | ARIMA |
| Non-Hispanic Black or African American | 2038 | 5.46 (0.67–10.25) | ARIMA |
| Non-Hispanic Black or African American | 2039 | 5.61 (0.42–10.81) | ARIMA |
| Non-Hispanic Black or African American | 2040 | 5.77 (0.16–11.38) | ARIMA |
| Non-Hispanic White | 2025 | 2.21 (1.77–2.66) | ETS |
| Non-Hispanic White | 2026 | 2.28 (1.65–2.92) | ETS |
| Non-Hispanic White | 2027 | 2.35 (1.47–3.23) | ETS |
| Non-Hispanic White | 2028 | 2.42 (1.25–3.59) | ETS |
| Non-Hispanic White | 2029 | 2.49 (0.99–3.98) | ETS |
| Non-Hispanic White | 2030 | 2.56 (0.70–4.41) | ETS |
| Non-Hispanic White | 2031 | 2.62 (0.37–4.88) | ETS |
| Non-Hispanic White | 2032 | 2.69 (0.01–5.38) | ETS |
| Non-Hispanic White | 2033 | 2.76 (0.00–5.91) | ETS |
| Non-Hispanic White | 2034 | 2.83 (0.00–6.48) | ETS |
| Non-Hispanic White | 2035 | 2.90 (0.00–7.09) | ETS |
| Non-Hispanic White | 2036 | 2.97 (0.00–7.73) | ETS |
| Non-Hispanic White | 2037 | 3.03 (0.00–8.41) | ETS |
| Non-Hispanic White | 2038 | 3.10 (0.00–9.13) | ETS |
| Non-Hispanic White | 2039 | 3.17 (0.00–9.90) | ETS |
| Non-Hispanic White | 2040 | 3.24 (0.00–10.71) | ETS |

*Abbreviations: AAMR, age-adjusted mortality rate; ARIMA, autoregressive integrated moving average; CI, confidence interval; ETS, error, trend, seasonal model. Note: Forecasted AAMRs are model-based projections and are reported per 100,000 population.*

**Supplementary Table 7. Annual mortality trends and age-adjusted mortality rates by US Census region, 1999-2024.**

| **US Census Region** | **Year** | **Deaths** | **Population** | **Age-adjusted mortality rate per 100,000 (95% CI)** |
| --- | --- | --- | --- | --- |
| Northeast | 1999 | 387 | 35633134 | 1.04 (0.94–1.14) |
| Northeast | 2000 | 355 | 35788687 | 0.93 (0.84–1.03) |
| Northeast | 2001 | 363 | 36006250 | 0.94 (0.85–1.04) |
| Northeast | 2002 | 312 | 36185082 | 0.80 (0.71–0.89) |
| Northeast | 2003 | 282 | 36346948 | 0.72 (0.64–0.81) |
| Northeast | 2004 | 289 | 36462699 | 0.73 (0.65–0.82) |
| Northeast | 2005 | 260 | 36559788 | 0.64 (0.57–0.72) |
| Northeast | 2006 | 280 | 36682176 | 0.69 (0.61–0.77) |
| Northeast | 2007 | 237 | 36846338 | 0.58 (0.51–0.66) |
| Northeast | 2008 | 229 | 37084149 | 0.55 (0.48–0.62) |
| Northeast | 2009 | 221 | 37339597 | 0.52 (0.45–0.59) |
| Northeast | 2010 | 266 | 37543347 | 0.62 (0.55–0.70) |
| Northeast | 2011 | 255 | 37864117 | 0.61 (0.53–0.68) |
| Northeast | 2012 | 223 | 38158527 | 0.49 (0.43–0.56) |
| Northeast | 2013 | 252 | 38437194 | 0.54 (0.47–0.61) |
| Northeast | 2014 | 237 | 38710627 | 0.52 (0.46–0.59) |
| Northeast | 2015 | 299 | 38965872 | 0.66 (0.58–0.73) |
| Northeast | 2016 | 351 | 39040202 | 0.76 (0.68–0.84) |
| Northeast | 2017 | 378 | 39417175 | 0.78 (0.70–0.86) |
| Northeast | 2018 | 386 | 39321978 | 0.79 (0.71–0.87) |
| Northeast | 2019 | 438 | 39381333 | 0.89 (0.81–0.98) |
| Northeast | 2020 | 503 | 39418559 | 0.98 (0.89–1.06) |
| Northeast | 2021 | 530 | 39700116 | 1.07 (0.98–1.17) |
| Northeast | 2022 | 504 | 39623477 | 0.96 (0.87–1.05) |
| Northeast | 2023 | 570 | 39713016 | 1.11 (1.02–1.21) |
| Northeast | 2024 | 685 | 40298417 | 1.27 (1.17–1.37) |
| Midwest | 1999 | 500 | 41293967 | 1.16 (1.06–1.26) |
| Midwest | 2000 | 499 | 41504992 | 1.16 (1.05–1.26) |
| Midwest | 2001 | 507 | 41762990 | 1.18 (1.08–1.28) |
| Midwest | 2002 | 472 | 41970731 | 1.08 (0.98–1.18) |
| Midwest | 2003 | 431 | 42200881 | 0.98 (0.89–1.08) |
| Midwest | 2004 | 410 | 42455980 | 0.94 (0.85–1.03) |
| Midwest | 2005 | 415 | 42748579 | 0.93 (0.84–1.02) |
| Midwest | 2006 | 346 | 43084311 | 0.77 (0.69–0.85) |
| Midwest | 2007 | 395 | 43424366 | 0.85 (0.77–0.94) |
| Midwest | 2008 | 302 | 43718509 | 0.65 (0.58–0.73) |
| Midwest | 2009 | 346 | 44010460 | 0.74 (0.66–0.82) |
| Midwest | 2010 | 361 | 44248465 | 0.75 (0.67–0.83) |
| Midwest | 2011 | 393 | 44584105 | 0.82 (0.74–0.90) |
| Midwest | 2012 | 390 | 44817227 | 0.76 (0.69–0.84) |
| Midwest | 2013 | 403 | 45090597 | 0.79 (0.71–0.86) |
| Midwest | 2014 | 501 | 45360409 | 0.97 (0.88–1.05) |
| Midwest | 2015 | 551 | 45628315 | 1.04 (0.95–1.13) |
| Midwest | 2016 | 653 | 45802491 | 1.21 (1.12–1.31) |
| Midwest | 2017 | 813 | 46143783 | 1.46 (1.36–1.56) |
| Midwest | 2018 | 942 | 46405110 | 1.66 (1.56–1.77) |
| Midwest | 2019 | 948 | 46589364 | 1.65 (1.54–1.76) |
| Midwest | 2020 | 1079 | 46721679 | 1.84 (1.73–1.95) |
| Midwest | 2021 | 1176 | 46391033 | 2.09 (1.97–2.22) |
| Midwest | 2022 | 1178 | 46415380 | 2.00 (1.89–2.12) |
| Midwest | 2023 | 1194 | 46614387 | 2.02 (1.91–2.14) |
| Midwest | 2024 | 1389 | 47151894 | 2.29 (2.17–2.42) |
| South | 1999 | 762 | 64108630 | 1.24 (1.15–1.33) |
| South | 2000 | 729 | 64843390 | 1.16 (1.08–1.25) |
| South | 2001 | 628 | 65874012 | 0.99 (0.91–1.06) |
| South | 2002 | 643 | 66758178 | 0.98 (0.90–1.05) |
| South | 2003 | 566 | 67639133 | 0.85 (0.78–0.92) |
| South | 2004 | 581 | 68718205 | 0.87 (0.80–0.94) |
| South | 2005 | 563 | 69951038 | 0.83 (0.76–0.89) |
| South | 2006 | 540 | 71198336 | 0.77 (0.71–0.84) |
| South | 2007 | 619 | 72387467 | 0.87 (0.80–0.94) |
| South | 2008 | 558 | 73529724 | 0.77 (0.70–0.83) |
| South | 2009 | 656 | 74596130 | 0.88 (0.81–0.94) |
| South | 2010 | 681 | 75419767 | 0.90 (0.83–0.97) |
| South | 2011 | 705 | 76650163 | 0.87 (0.80–0.93) |
| South | 2012 | 805 | 77681893 | 1.00 (0.93–1.06) |
| South | 2013 | 899 | 78693557 | 1.06 (0.99–1.13) |
| South | 2014 | 961 | 79945474 | 1.10 (1.03–1.17) |
| South | 2015 | 1092 | 81260812 | 1.22 (1.14–1.29) |
| South | 2016 | 1351 | 82405493 | 1.48 (1.40–1.56) |
| South | 2017 | 1513 | 83659214 | 1.60 (1.52–1.68) |
| South | 2018 | 1687 | 84717024 | 1.73 (1.65–1.82) |
| South | 2019 | 1829 | 85606773 | 1.83 (1.74–1.91) |
| South | 2020 | 1865 | 86611804 | 1.79 (1.71–1.87) |
| South | 2021 | 1936 | 85612869 | 1.96 (1.88–2.05) |
| South | 2022 | 2092 | 86583051 | 2.00 (1.91–2.08) |
| South | 2023 | 2191 | 87764878 | 2.07 (1.98–2.16) |
| South | 2024 | 2365 | 89589072 | 2.15 (2.06–2.24) |
| West | 1999 | 300 | 39373038 | 0.85 (0.75–0.95) |
| West | 2000 | 284 | 39847571 | 0.82 (0.72–0.91) |
| West | 2001 | 283 | 40661876 | 0.78 (0.69–0.88) |
| West | 2002 | 270 | 41294037 | 0.74 (0.65–0.83) |
| West | 2003 | 274 | 41903467 | 0.72 (0.64–0.81) |
| West | 2004 | 263 | 42568500 | 0.68 (0.60–0.76) |
| West | 2005 | 329 | 43291979 | 0.84 (0.75–0.93) |
| West | 2006 | 285 | 44054536 | 0.72 (0.63–0.80) |
| West | 2007 | 322 | 44745606 | 0.79 (0.70–0.87) |
| West | 2008 | 320 | 45462708 | 0.74 (0.66–0.82) |
| West | 2009 | 381 | 46160829 | 0.88 (0.79–0.97) |
| West | 2010 | 463 | 46680404 | 1.02 (0.93–1.12) |
| West | 2011 | 523 | 47494551 | 1.13 (1.03–1.22) |
| West | 2012 | 469 | 48168390 | 0.99 (0.90–1.08) |
| West | 2013 | 573 | 48863966 | 1.18 (1.08–1.28) |
| West | 2014 | 638 | 49792770 | 1.25 (1.15–1.34) |
| West | 2015 | 734 | 50698818 | 1.37 (1.27–1.47) |
| West | 2016 | 996 | 51393231 | 1.81 (1.69–1.92) |
| West | 2017 | 1061 | 52227159 | 1.89 (1.77–2.00) |
| West | 2018 | 1263 | 52867078 | 2.20 (2.08–2.32) |
| West | 2019 | 1316 | 53403697 | 2.22 (2.10–2.35) |
| West | 2020 | 1417 | 53882971 | 2.32 (2.19–2.44) |
| West | 2021 | 1407 | 52326577 | 2.42 (2.30–2.55) |
| West | 2022 | 1515 | 52503527 | 2.48 (2.36–2.61) |
| West | 2023 | 1596 | 52903171 | 2.58 (2.46–2.72) |
| West | 2024 | 1644 | 53792507 | 2.53 (2.41–2.66) |

*Abbreviations: AAMR, age-adjusted mortality rate; CI, confidence interval. Note: AAMRs are reported per 100,000 population.*

**Supplementary Table 8. Projected age-adjusted mortality rates by US Census region, 2025-2040.**

| **Census** | **Year** | **Age-adjusted mortality rate per 100,000 (95% CI)** | **Forecast model** |
| --- | --- | --- | --- |
| Northeast | 2025 | 1.33 (1.15–1.51) | ETS |
| Northeast | 2026 | 1.40 (1.18–1.61) | ETS |
| Northeast | 2027 | 1.46 (1.19–1.73) | ETS |
| Northeast | 2028 | 1.52 (1.20–1.85) | ETS |
| Northeast | 2029 | 1.59 (1.19–1.99) | ETS |
| Northeast | 2030 | 1.65 (1.18–2.13) | ETS |
| Northeast | 2031 | 1.72 (1.16–2.28) | ETS |
| Northeast | 2032 | 1.78 (1.13–2.43) | ETS |
| Northeast | 2033 | 1.85 (1.10–2.59) | ETS |
| Northeast | 2034 | 1.91 (1.07–2.76) | ETS |
| Northeast | 2035 | 1.98 (1.03–2.93) | ETS |
| Northeast | 2036 | 2.04 (0.98–3.10) | ETS |
| Northeast | 2037 | 2.11 (0.93–3.28) | ETS |
| Northeast | 2038 | 2.17 (0.88–3.46) | ETS |
| Northeast | 2039 | 2.23 (0.82–3.65) | ETS |
| Northeast | 2040 | 2.30 (0.76–3.84) | ETS |
| Midwest | 2025 | 2.34 (2.03–2.65) | ETS |
| Midwest | 2026 | 2.42 (1.99–2.85) | ETS |
| Midwest | 2027 | 2.49 (1.91–3.08) | ETS |
| Midwest | 2028 | 2.57 (1.80–3.34) | ETS |
| Midwest | 2029 | 2.65 (1.68–3.62) | ETS |
| Midwest | 2030 | 2.72 (1.53–3.91) | ETS |
| Midwest | 2031 | 2.80 (1.37–4.23) | ETS |
| Midwest | 2032 | 2.87 (1.19–4.56) | ETS |
| Midwest | 2033 | 2.95 (1.00–4.90) | ETS |
| Midwest | 2034 | 3.03 (0.79–5.26) | ETS |
| Midwest | 2035 | 3.10 (0.57–5.63) | ETS |
| Midwest | 2036 | 3.18 (0.34–6.02) | ETS |
| Midwest | 2037 | 3.25 (0.10–6.41) | ETS |
| Midwest | 2038 | 3.33 (0.00–6.82) | ETS |
| Midwest | 2039 | 3.41 (0.00–7.24) | ETS |
| Midwest | 2040 | 3.48 (0.00–7.67) | ETS |
| South | 2025 | 2.26 (2.01–2.51) | ARIMA |
| South | 2026 | 2.25 (1.88–2.63) | ARIMA |
| South | 2027 | 2.33 (1.78–2.88) | ARIMA |
| South | 2028 | 2.38 (1.69–3.08) | ARIMA |
| South | 2029 | 2.41 (1.51–3.30) | ARIMA |
| South | 2030 | 2.50 (1.40–3.59) | ARIMA |
| South | 2031 | 2.51 (1.21–3.81) | ARIMA |
| South | 2032 | 2.58 (1.05–4.11) | ARIMA |
| South | 2033 | 2.63 (0.87–4.38) | ARIMA |
| South | 2034 | 2.67 (0.66–4.68) | ARIMA |
| South | 2035 | 2.74 (0.47–5.00) | ARIMA |
| South | 2036 | 2.77 (0.23–5.30) | ARIMA |
| South | 2037 | 2.83 (0.02–5.64) | ARIMA |
| South | 2038 | 2.87 (0.00–5.97) | ARIMA |
| South | 2039 | 2.92 (0.00–6.32) | ARIMA |
| South | 2040 | 2.98 (0.00–6.68) | ARIMA |
| West | 2025 | 2.66 (2.28–3.03) | ETS |
| West | 2026 | 2.70 (2.18–3.22) | ETS |
| West | 2027 | 2.75 (2.07–3.42) | ETS |
| West | 2028 | 2.79 (1.94–3.64) | ETS |
| West | 2029 | 2.84 (1.80–3.87) | ETS |
| West | 2030 | 2.88 (1.65–4.12) | ETS |
| West | 2031 | 2.93 (1.48–4.38) | ETS |
| West | 2032 | 2.97 (1.30–4.65) | ETS |
| West | 2033 | 3.02 (1.11–4.92) | ETS |
| West | 2034 | 3.06 (0.91–5.21) | ETS |
| West | 2035 | 3.11 (0.70–5.51) | ETS |
| West | 2036 | 3.15 (0.48–5.82) | ETS |
| West | 2037 | 3.20 (0.26–6.14) | ETS |
| West | 2038 | 3.24 (0.02–6.47) | ETS |
| West | 2039 | 3.29 (0.00–6.80) | ETS |
| West | 2040 | 3.33 (0.00–7.15) | ETS |

*Abbreviations: AAMR, age-adjusted mortality rate; ARIMA, autoregressive integrated moving average; CI, confidence interval; ETS, error, trend, seasonal model. Note: Forecasted AAMRs are model-based projections and are reported per 100,000 population.*

**Supplementary Table 9. Annual mortality trends and age-adjusted mortality rates by urban classification, 1999-2020.**

| **Urban** | **Year** | **Deaths** | **Population** | **Age-adjusted mortality rate per 100,000 (95% CI)** |
| --- | --- | --- | --- | --- |
| Metropolitan | 1999 | 1542 | 151245342 | 1.07 (1.02–1.13) |
| Metropolitan | 2000 | 1465 | 152658699 | 1.02 (0.97–1.08) |
| Metropolitan | 2001 | 1382 | 154896258 | 0.95 (0.90–1.00) |
| Metropolitan | 2002 | 1313 | 156659051 | 0.87 (0.83–0.92) |
| Metropolitan | 2003 | 1204 | 158367715 | 0.80 (0.76–0.85) |
| Metropolitan | 2004 | 1195 | 160272097 | 0.78 (0.74–0.83) |
| Metropolitan | 2005 | 1233 | 162371826 | 0.78 (0.74–0.83) |
| Metropolitan | 2006 | 1165 | 164523389 | 0.74 (0.69–0.78) |
| Metropolitan | 2007 | 1257 | 166650886 | 0.79 (0.74–0.83) |
| Metropolitan | 2008 | 1140 | 168826027 | 0.69 (0.65–0.73) |
| Metropolitan | 2009 | 1269 | 170965574 | 0.74 (0.70–0.79) |
| Metropolitan | 2010 | 1459 | 172591105 | 0.84 (0.80–0.89) |
| Metropolitan | 2011 | 1516 | 175204532 | 0.85 (0.81–0.89) |
| Metropolitan | 2012 | 1520 | 177423676 | 0.83 (0.79–0.87) |
| Metropolitan | 2013 | 1705 | 179634449 | 0.89 (0.85–0.93) |
| Metropolitan | 2014 | 1874 | 182304016 | 0.97 (0.93–1.02) |
| Metropolitan | 2015 | 2172 | 184959306 | 1.10 (1.05–1.14) |
| Metropolitan | 2016 | 2761 | 186963190 | 1.35 (1.30–1.40) |
| Metropolitan | 2017 | 3115 | 189675062 | 1.48 (1.43–1.53) |
| Metropolitan | 2018 | 3501 | 191415024 | 1.62 (1.56–1.67) |
| Metropolitan | 2019 | 3703 | 193006488 | 1.65 (1.60–1.70) |
| Metropolitan | 2020 | 3979 | 194600110 | 1.73 (1.68–1.79) |
| Non-metropolitan | 1999 | 407 | 29163427 | 1.19 (1.08–1.31) |
| Non-metropolitan | 2000 | 402 | 29325941 | 1.18 (1.06–1.29) |
| Non-metropolitan | 2001 | 399 | 29408870 | 1.15 (1.04–1.26) |
| Non-metropolitan | 2002 | 384 | 29548977 | 1.11 (1.00–1.22) |
| Non-metropolitan | 2003 | 349 | 29722714 | 0.99 (0.89–1.10) |
| Non-metropolitan | 2004 | 348 | 29933287 | 0.99 (0.88–1.09) |
| Non-metropolitan | 2005 | 334 | 30179558 | 0.93 (0.83–1.03) |
| Non-metropolitan | 2006 | 286 | 30495970 | 0.79 (0.70–0.88) |
| Non-metropolitan | 2007 | 316 | 30752891 | 0.87 (0.77–0.97) |
| Non-metropolitan | 2008 | 269 | 30969063 | 0.71 (0.63–0.80) |
| Non-metropolitan | 2009 | 335 | 31141442 | 0.90 (0.80–0.99) |
| Non-metropolitan | 2010 | 312 | 31300878 | 0.82 (0.73–0.91) |
| Non-metropolitan | 2011 | 360 | 31388404 | 0.92 (0.82–1.02) |
| Non-metropolitan | 2012 | 367 | 31402361 | 0.94 (0.84–1.03) |
| Non-metropolitan | 2013 | 422 | 31450865 | 1.04 (0.94–1.14) |
| Non-metropolitan | 2014 | 463 | 31505264 | 1.14 (1.04–1.25) |
| Non-metropolitan | 2015 | 504 | 31594511 | 1.21 (1.10–1.31) |
| Non-metropolitan | 2016 | 590 | 31678227 | 1.40 (1.29–1.52) |
| Non-metropolitan | 2017 | 650 | 31772269 | 1.50 (1.38–1.62) |
| Non-metropolitan | 2018 | 777 | 31896166 | 1.78 (1.65–1.90) |
| Non-metropolitan | 2019 | 828 | 31974679 | 1.85 (1.72–1.97) |
| Non-metropolitan | 2020 | 885 | 32028405 | 1.94 (1.81–2.07) |

*Abbreviations: AAMR, age-adjusted mortality rate; CI, confidence interval. Note: Urban classification data were available through 2020; therefore, this table covers 1999-2020.*

**Supplementary Table 10. Projected age-adjusted mortality rates by urban classification, 2021-2040.**

| **Urban** | **Year** | **Age-adjusted mortality rate per 100,000 (95% CI)** | **Forecast model** |
| --- | --- | --- | --- |
| Metropolitan | 2021 | 1.82 (1.67–1.96) | ETS |
| Metropolitan | 2022 | 1.90 (1.65–2.16) | ETS |
| Metropolitan | 2023 | 1.99 (1.61–2.36) | ETS |
| Metropolitan | 2024 | 2.08 (1.57–2.58) | ETS |
| Metropolitan | 2025 | 2.16 (1.51–2.81) | ETS |
| Metropolitan | 2026 | 2.25 (1.45–3.05) | ETS |
| Metropolitan | 2027 | 2.34 (1.37–3.30) | ETS |
| Metropolitan | 2028 | 2.42 (1.28–3.56) | ETS |
| Metropolitan | 2029 | 2.51 (1.19–3.83) | ETS |
| Metropolitan | 2030 | 2.60 (1.08–4.11) | ETS |
| Metropolitan | 2031 | 2.68 (0.97–4.39) | ETS |
| Metropolitan | 2032 | 2.77 (0.85–4.69) | ETS |
| Metropolitan | 2033 | 2.86 (0.72–4.99) | ETS |
| Metropolitan | 2034 | 2.94 (0.59–5.30) | ETS |
| Metropolitan | 2035 | 3.03 (0.45–5.61) | ETS |
| Metropolitan | 2036 | 3.12 (0.30–5.93) | ETS |
| Metropolitan | 2037 | 3.20 (0.14–6.26) | ETS |
| Metropolitan | 2038 | 3.29 (0.00–6.60) | ETS |
| Metropolitan | 2039 | 3.37 (0.00–6.94) | ETS |
| Metropolitan | 2040 | 3.46 (0.00–7.29) | ETS |
| Non-metropolitan | 2021 | 2.09 (1.94–2.25) | ARIMA |
| Non-metropolitan | 2022 | 2.16 (1.96–2.37) | ARIMA |
| Non-metropolitan | 2023 | 2.31 (1.98–2.63) | ARIMA |
| Non-metropolitan | 2024 | 2.40 (1.97–2.83) | ARIMA |
| Non-metropolitan | 2025 | 2.52 (1.96–3.08) | ARIMA |
| Non-metropolitan | 2026 | 2.63 (1.93–3.33) | ARIMA |
| Non-metropolitan | 2027 | 2.74 (1.89–3.58) | ARIMA |
| Non-metropolitan | 2028 | 2.85 (1.85–3.86) | ARIMA |
| Non-metropolitan | 2029 | 2.96 (1.79–4.13) | ARIMA |
| Non-metropolitan | 2030 | 3.08 (1.73–4.42) | ARIMA |
| Non-metropolitan | 2031 | 3.19 (1.66–4.71) | ARIMA |
| Non-metropolitan | 2032 | 3.30 (1.58–5.01) | ARIMA |
| Non-metropolitan | 2033 | 3.41 (1.50–5.32) | ARIMA |
| Non-metropolitan | 2034 | 3.52 (1.41–5.64) | ARIMA |
| Non-metropolitan | 2035 | 3.63 (1.31–5.96) | ARIMA |
| Non-metropolitan | 2036 | 3.74 (1.20–6.29) | ARIMA |
| Non-metropolitan | 2037 | 3.86 (1.09–6.62) | ARIMA |
| Non-metropolitan | 2038 | 3.97 (0.97–6.97) | ARIMA |
| Non-metropolitan | 2039 | 4.08 (0.84–7.31) | ARIMA |
| Non-metropolitan | 2040 | 4.19 (0.71–7.67) | ARIMA |

*Abbreviations: AAMR, age-adjusted mortality rate; ARIMA, autoregressive integrated moving average; CI, confidence interval; ETS, error, trend, seasonal model. Note: Urban classification projections begin in 2021 because observed urban classification data were available through 2020. Forecasted AAMRs are model-based projections and are reported per 100,000 population.*

**Supplementary Table 11. State-specific mortality burden and age-adjusted mortality rates, 2021-2024.**

| **State** | **State code** | **Deaths, n** | **Population** | **Crude rate (95% CI)** | **AAMR (95% CI)** | **Percentage, %** |
| --- | --- | --- | --- | --- | --- | --- |
| Alabama | 1 | 223 | 13,797,763 | 1.62 (1.4-1.83) | 1.29 (1.13-1.49) | 1.01 |
| Alaska | 2 | 19 | 1,866,059 | 1.02 (0.61-1.59) | 1.13 (0.66-1.83) | 0.09 |
| Arizona | 4 | 493 | 20,017,546 | 2.46 (2.25-2.68) | 1.84 (1.68-2.02) | 2.24 |
| Arkansas | 5 | 303 | 8,149,451 | 3.72 (3.3-4.14) | 2.9 (2.58-3.26) | 1.38 |
| California | 6 | 2,735 | 104,883,987 | 2.61 (2.51-2.71) | 2.28 (2.2-2.37) | 12.45 |
| Colorado | 8 | 370 | 16,052,602 | 2.3 (2.07-2.54) | 2.21 (1.99-2.46) | 1.68 |
| Connecticut | 9 | 167 | 10,065,743 | 1.66 (1.41-1.91) | 1.27 (1.08-1.48) | 0.76 |
| Delaware | 10 | 89 | 2,865,863 | 3.11 (2.49-3.82) | 2.26 (1.81-2.82) | 0.41 |
| District of Columbia | 11 | 17 | 1,897,260 | 0.9 (0.52-1.43) | 0.93 (0.54-1.52) | 0.08 |
| Florida | 12 | 1,935 | 64,230,389 | 3.01 (2.88-3.15) | 2.04 (1.95-2.14) | 8.81 |
| Georgia | 13 | 541 | 29,161,084 | 1.86 (1.7-2.01) | 1.72 (1.57-1.87) | 2.46 |
| Hawaii | 15 | 191 | 3,339,167 | 5.72 (4.91-6.53) | 3.57 (3.07-4.14) | 0.87 |
| Idaho | 16 | 184 | 5,076,481 | 3.62 (3.1-4.15) | 3.1 (2.66-3.6) | 0.84 |
| Illinois | 17 | 533 | 34,482,696 | 1.55 (1.41-1.68) | 1.27 (1.16-1.38) | 2.43 |
| Indiana | 18 | 638 | 18,183,416 | 3.51 (3.24-3.78) | 2.9 (2.67-3.14) | 2.90 |
| Iowa | 19 | 329 | 8,533,257 | 3.86 (3.44-4.27) | 2.85 (2.54-3.18) | 1.50 |
| Kansas | 20 | 220 | 7,645,294 | 2.88 (2.5-3.26) | 2.26 (1.97-2.59) | 1.00 |
| Kentucky | 21 | 395 | 12,262,340 | 3.22 (2.9-3.54) | 2.71 (2.44-3) | 1.80 |
| Louisiana | 22 | 122 | 12,280,517 | 0.99 (0.82-1.17) | 0.82 (0.68-0.99) | 0.56 |
| Maine | 23 | 87 | 4,069,412 | 2.14 (1.71-2.64) | 1.54 (1.23-1.93) | 0.40 |
| Maryland | 24 | 447 | 16,874,606 | 2.65 (2.4-2.89) | 2.25 (2.04-2.47) | 2.03 |
| Massachusetts | 25 | 242 | 19,577,088 | 1.24 (1.08-1.39) | 1 (0.88-1.14) | 1.10 |
| Michigan | 26 | 635 | 27,572,232 | 2.3 (2.12-2.48) | 1.81 (1.67-1.96) | 2.89 |
| Minnesota | 27 | 495 | 15,466,059 | 3.2 (2.92-3.48) | 2.59 (2.36-2.83) | 2.25 |
| Mississippi | 28 | 236 | 7,839,974 | 3.01 (2.63-3.39) | 2.44 (2.14-2.78) | 1.07 |
| Missouri | 29 | 322 | 16,753,129 | 1.92 (1.71-2.13) | 1.5 (1.34-1.68) | 1.47 |
| Montana | 30 | 55 | 3,085,385 | 1.78 (1.34-2.32) | 1.32 (1-1.76) | 0.25 |
| Nebraska | 31 | 187 | 5,131,723 | 3.64 (3.12-4.17) | 2.93 (2.52-3.39) | 0.85 |
| Nevada | 32 | 447 | 8,689,004 | 5.14 (4.67-5.62) | 4.52 (4.11-4.98) | 2.03 |
| New Hampshire | 33 | 80 | 4,051,613 | 1.97 (1.57-2.46) | 1.52 (1.2-1.92) | 0.36 |
| New Jersey | 34 | 192 | 25,678,886 | 0.75 (0.64-0.85) | 0.6 (0.52-0.69) | 0.87 |
| New Mexico | 35 | 111 | 5,717,738 | 1.94 (1.58-2.3) | 1.44 (1.19-1.75) | 0.51 |
| New York | 36 | 775 | 54,737,782 | 1.42 (1.32-1.52) | 1.1 (1.02-1.18) | 3.53 |
| North Carolina | 37 | 580 | 29,287,109 | 1.98 (1.82-2.14) | 1.67 (1.53-1.81) | 2.64 |
| North Dakota | 38 | 38 | 2,016,127 | 1.88 (1.33-2.59) | 1.44 (1.01-2.02) | 0.17 |
| Ohio | 39 | 1,142 | 32,177,983 | 3.55 (3.34-3.75) | 2.78 (2.61-2.95) | 5.20 |
| Oklahoma | 40 | 166 | 10,230,058 | 1.62 (1.38-1.87) | 1.34 (1.14-1.57) | 0.76 |
| Oregon | 41 | 726 | 11,822,408 | 6.14 (5.69-6.59) | 4.86 (4.51-5.23) | 3.30 |
| Pennsylvania | 42 | 608 | 36,237,944 | 1.68 (1.54-1.81) | 1.23 (1.13-1.34) | 2.77 |
| Rhode Island | 44 | 28 | 3,073,059 | 0.91 (0.61-1.32) | 0.69 (0.46-1.03) | 0.13 |
| South Carolina | 45 | 558 | 14,667,964 | 3.8 (3.49-4.12) | 3.02 (2.77-3.29) | 2.54 |
| South Dakota | 46 | 35 | 2,388,439 | 1.47 (1.02-2.04) | 1.13 (0.78-1.61) | 0.16 |
| Tennessee | 47 | 688 | 19,347,331 | 3.56 (3.29-3.82) | 3.04 (2.81-3.28) | 3.13 |
| Texas | 48 | 1,729 | 78,065,388 | 2.21 (2.11-2.32) | 2.24 (2.13-2.34) | 7.87 |
| Utah | 49 | 102 | 8,140,519 | 1.25 (1.01-1.5) | 1.35 (1.09-1.64) | 0.46 |
| Vermont | 50 | 110 | 1,843,499 | 5.97 (4.85-7.08) | 4.23 (3.46-5.16) | 0.50 |
| Virginia | 51 | 424 | 23,590,932 | 1.8 (1.63-1.97) | 1.54 (1.39-1.69) | 1.93 |
| Washington | 53 | 688 | 21,263,295 | 3.24 (2.99-3.48) | 2.84 (2.63-3.07) | 3.13 |
| West Virginia | 54 | 131 | 5,001,841 | 2.62 (2.17-3.07) | 1.88 (1.56-2.25) | 0.60 |
| Wisconsin | 55 | 363 | 16,222,339 | 2.24 (2.01-2.47) | 1.73 (1.55-1.92) | 1.65 |
| Wyoming | 56 | 41 | 1,571,591 | 2.61 (1.87-3.54) | 2.13 (1.52-2.95) | 0.19 |
| **Total** |  | **21,972** | **906,983,372** | **2.42 (2.39-2.45)** | **2 (1.97-2.02)** | **100.00** |

*Abbreviations: AAMR, age-adjusted mortality rate; CI, confidence interval. Note: State-level results use the CDC WONDER Multiple Cause of Death 2018-2024 file queried for 2021-2024 and include the District of Columbia. Rates are per 100,000 population and were age-adjusted to the 2000 US standard population. Percentages are based on 21,972 state-classified deaths; percentages may not sum to 100.00 because of rounding.*

**Supplementary Table 12. Distribution of mortality by place of death, 1999-2020.**

| **Place of death** | **Place of death code** | **Deaths, n** | **Percentage, %** |
| --- | --- | --- | --- |
| Decedent's home | 4 | 24,962 | 48.51 |
| Nursing home/long-term care | 6 | 9,496 | 18.45 |
| Medical facility - inpatient | 1 | 8,185 | 15.91 |
| Hospice facility | 5 | 5,386 | 10.47 |
| Other | 7 | 2,698 | 5.24 |
| Medical facility - outpatient or ER | 2 | 594 | 1.15 |
| Place of death unknown | 9 | 67 | 0.13 |
| Medical facility - dead on arrival | 3 | 42 | 0.08 |
| Medical facility - status unknown | 10 | 27 | 0.05 |
| **Total** |  | **51,457** | **100.00** |

*Note: Percentages were calculated using 51,457 deaths with place-of-death data available for 1999-2020. Percentages may not sum to exactly 100.00 because of rounding*

**Supplementary Table 13. Annual mortality trends and age-adjusted mortality rates for prostate cancer as the underlying cause of death, 1999-2024.**

| **Cause** | **Year** | **Deaths** | **Population** | **Age-adjusted mortality rate per 100,000 (95% CI)** |
| --- | --- | --- | --- | --- |
| UCD PC | 1999 | 31661 | 180408769 | 17.96 (17.76–18.16) |
| UCD PC | 2000 | 30997 | 181984640 | 17.39 (17.20–17.58) |
| UCD PC | 2001 | 30632 | 184305128 | 16.91 (16.73–17.10) |
| UCD PC | 2002 | 30359 | 186208028 | 16.55 (16.36–16.73) |
| UCD PC | 2003 | 29496 | 188090429 | 15.82 (15.64–16.00) |
| UCD PC | 2004 | 28958 | 190205384 | 15.32 (15.14–15.50) |
| UCD PC | 2005 | 28864 | 192551384 | 14.98 (14.81–15.16) |
| UCD PC | 2006 | 28317 | 195019359 | 14.41 (14.24–14.58) |
| UCD PC | 2007 | 29063 | 197403777 | 14.52 (14.35–14.69) |
| UCD PC | 2008 | 28414 | 199795090 | 13.92 (13.75–14.08) |
| UCD PC | 2009 | 28040 | 202107016 | 13.45 (13.29–13.61) |
| UCD PC | 2010 | 28505 | 203891983 | 13.44 (13.28–13.59) |
| UCD PC | 2011 | 27918 | 206592936 | 12.79 (12.63–12.94) |
| UCD PC | 2012 | 27187 | 208826037 | 12.17 (12.02–12.31) |
| UCD PC | 2013 | 27614 | 211085314 | 11.99 (11.85–12.14) |
| UCD PC | 2014 | 28242 | 213809280 | 11.99 (11.85–12.13) |
| UCD PC | 2015 | 28775 | 216553817 | 11.92 (11.78–12.06) |
| UCD PC | 2016 | 30289 | 218641417 | 12.25 (12.11–12.39) |
| UCD PC | 2017 | 30412 | 221447331 | 12.00 (11.87–12.14) |
| UCD PC | 2018 | 31411 | 223311190 | 12.10 (11.96–12.23) |
| UCD PC | 2019 | 31569 | 224981167 | 11.84 (11.71–11.98) |
| UCD PC | 2020 | 32644 | 226635013 | 11.99 (11.86–12.12) |
| UCD PC | 2021 | 32340 | 224030595 | 12.37 (12.24–12.51) |
| UCD PC | 2022 | 33153 | 225125435 | 12.03 (11.90–12.16) |
| UCD PC | 2023 | 33612 | 226995452 | 12.11 (11.98–12.24) |
| UCD PC | 2024 | 34560 | 230831890 | 12.05 (11.92–12.18) |

*Abbreviations: AAMR, age-adjusted mortality rate; CI, confidence interval; UCD PC, prostate cancer listed as the underlying cause of death. Note: AAMRs are reported per 100,000 population.*

*.*
